# Bidirectional regulation of motor circuits using magnetogenetic gene therapy

**DOI:** 10.1126/sciadv.adp9150

**Published:** 2024-10-09

**Authors:** Santiago R. Unda, Lisa E. Pomeranz, Roberta Marongiu, Xiaofei Yu, Leah Kelly, Gholamreza Hassanzadeh, Henrik Molina, George Vaisey, Putianqi Wang, Jonathan P. Dyke, Edward K. Fung, Logan Grosenick, Rick Zirkel, Aldana M. Antoniazzi, Sofya Norman, Conor M. Liston, Chris Schaffer, Nozomi Nishimura, Sarah A. Stanley, Jeffrey M. Friedman, Michael G. Kaplitt

**Affiliations:** ^1^ Laboratory of Molecular Neurosurgery, Department of Neurological Surgery, Weill Cornell Medical College, Cornell University, New York, NY 10065, USA.; ^2^ Laboratory of Molecular Genetics, Rockefeller University, New York, NY 10065, USA.; ^3^ School of Life Sciences, Fudan University, Shanghai 200433, China.; ^4^ VIB Nanobody Core, Vrije Universiteit Brussel, Pleinlaan 2, 1050 Brussels, Belgium.; ^5^ Diabetes, Obesity and Metabolism Institute, Icahn School of Medicine at Mount Sinai, New York, NY 10019, USA.; ^6^ Laboratory of Molecular Neurobiology and Biophysics, Rockefeller University, New York, NY 10065, USA.; ^7^ Citigroup Bioimaging Center, Weill Cornell Medical College, Cornell University, New York, NY 10065, USA.; ^8^ Department of Psychiatry, Weill Cornell Medical College, Cornell University, New York, NY 10065, USA.; ^9^ Meining School of Biomedical Engineering, Cornell University, Ithaca, NY 14850, USA.; ^10^ Nash Family Department of Neuroscience and Friedman Brain Institute, Icahn School of Medicine at Mount Sinai, New York, NY 10019, USA.; ^11^ Howard Hughes Medical Institute, The Rockefeller University, New York, NY 10065, USA.

## Abstract

Here, we report a magnetogenetic system, based on a single anti-ferritin nanobody-TRPV1 receptor fusion protein, which regulated neuronal activity when exposed to magnetic fields. Adeno-associated virus (AAV)–mediated delivery of a floxed nanobody-TRPV1 into the striatum of adenosine-2a receptor–Cre drivers resulted in motor freezing when placed in a magnetic resonance imaging machine or adjacent to a transcranial magnetic stimulation device. Functional imaging and fiber photometry confirmed activation in response to magnetic fields. Expression of the same construct in the striatum of wild-type mice along with a second injection of an AAVretro expressing Cre into the globus pallidus led to similar circuit specificity and motor responses. Last, a mutation was generated to gate chloride and inhibit neuronal activity. Expression of this variant in the subthalamic nucleus in PitX2-Cre parkinsonian mice resulted in reduced *c-fos* expression and motor rotational behavior. These data demonstrate that magnetogenetic constructs can bidirectionally regulate activity of specific neuronal circuits noninvasively in vivo using clinically available devices.

## Teaser

A gene therapy toolbox allows noninvasive magnetic field regulation of targeted neural circuit activity in vivo.

## INTRODUCTION

Neuromodulation has become a critical tool for both delineating functional brain circuits underlying behaviors in animal studies and treating human patients with circuit disorders (*1*). Electrical stimulation has long been used in preclinical studies, and deep brain stimulation (DBS) is a standard of care for patients with advanced tremors and Parkinson’s disease (PD) (*2*). Despite the focal effects of electrical stimulation, the need for greater precision in modulating specific circuits or neuronal populations has promoted the development of novel technologies. Optogenetics combines viral delivery of light-sensitive ion channels with regulated light probes for precise millisecond-timescale control of neural activity (*3*, *4*), but the microbial origin of the channels and the need for a fiber optic implant can limit applications in animal studies and provide challenges to human translation (*5*). In contrast, chemogenetics delivers genes for modified channels or receptors to allow drug-regulated control of neural activity without an implant (*6*), but the response is dependent on drug diffusion to targeted regions and the time course and longevity of responses rely on the pharmacokinetics of the drug.

Precise spatial and temporal control of neural circuitry without implanted devices could be transformative for both preclinical studies and human therapeutic applications (*7*). In vivo magnetic field–based stimulation to control neural activity has shown promise in mammalian (*5*, *7*–*11*) and nonmammalian systems (*7*, *12*). Electromagnetic fields can penetrate tissue and transmit energy to metal/metal oxide particles, which, in turn, gate receptors and ion channels (*13*). Taking advantage of these properties, regulation of several brain structures has been achieved using endogenous or genetically encoded members of the transient receptor potential cation channel subfamily V (TRPV). Tethering of exogenous ferritin or external iron oxide nanoparticles (*5*, *7*, *9*) has been shown to lead to alterations of neural activity by magnetic fields in vitro and in vivo. Previously, the use of this system required adeno-associated virus (AAV) vectors to introduce fusions of TRPV channels with exogenous subunits of paramagnetic ferritin into the cells or the use of adenovirus vectors to deliver TRPV1 and ferritin cDNA clones, which were too large for a single AAV vector.

Here, we report highly cell-specific, temporally precise, remote, and reversible modulation of deep brain structures in awake, freely moving mice using a novel nanobody (Nb) to endogenous ferritin (Ft) fused to the TRPV1 channel (Nb-Ft-TRPV1) encoded by a construct packaged into a single AAV vector. We show that Cre-dependent magnetogenetic activation of striatopallidal neurons elicits robust motor freezing in Adora-2a (A2a)–Cre mice, similar to that reported using opto- and chemogenetics (*14*–*16*). The magnetic fields were delivered using standard magnetic resonance imaging (MRI) machines or a commercially available transcranial magnetic stimulation (TMS) device used for human therapy, with a threshold of 180 mT to induce behavior alterations. Neural activation was further confirmed using expression of *c-fos*, positron emission tomography (PET), and in vivo fiber photometry to validate magnetogenetic-induced changes of neural activation. Circuit-specific activation of striatopallidal neurons was also achieved in wild-type (WT) mice using a retrograde AAV encoding Cre recombinase in combination with the Cre-conditional magnetogenetic construct. Last, we achieved motor improvements in parkinsonian mice using noninvasive, magnetogenetic inhibition of subthalamic nucleus (STN) neurons with a nanobody-TRPV1 construct modified to block neuronal activity. The approach that is presented provides for a means for inducing bidirectional, real-time regulation of neuronal circuits in freely moving animals in a magnetic field and may have potential for a variety of human therapeutic applications.

## RESULTS

### Generation and validation of ferritin-binding nanobodies for magnet stimulation in vitro

**Fig. 1.**
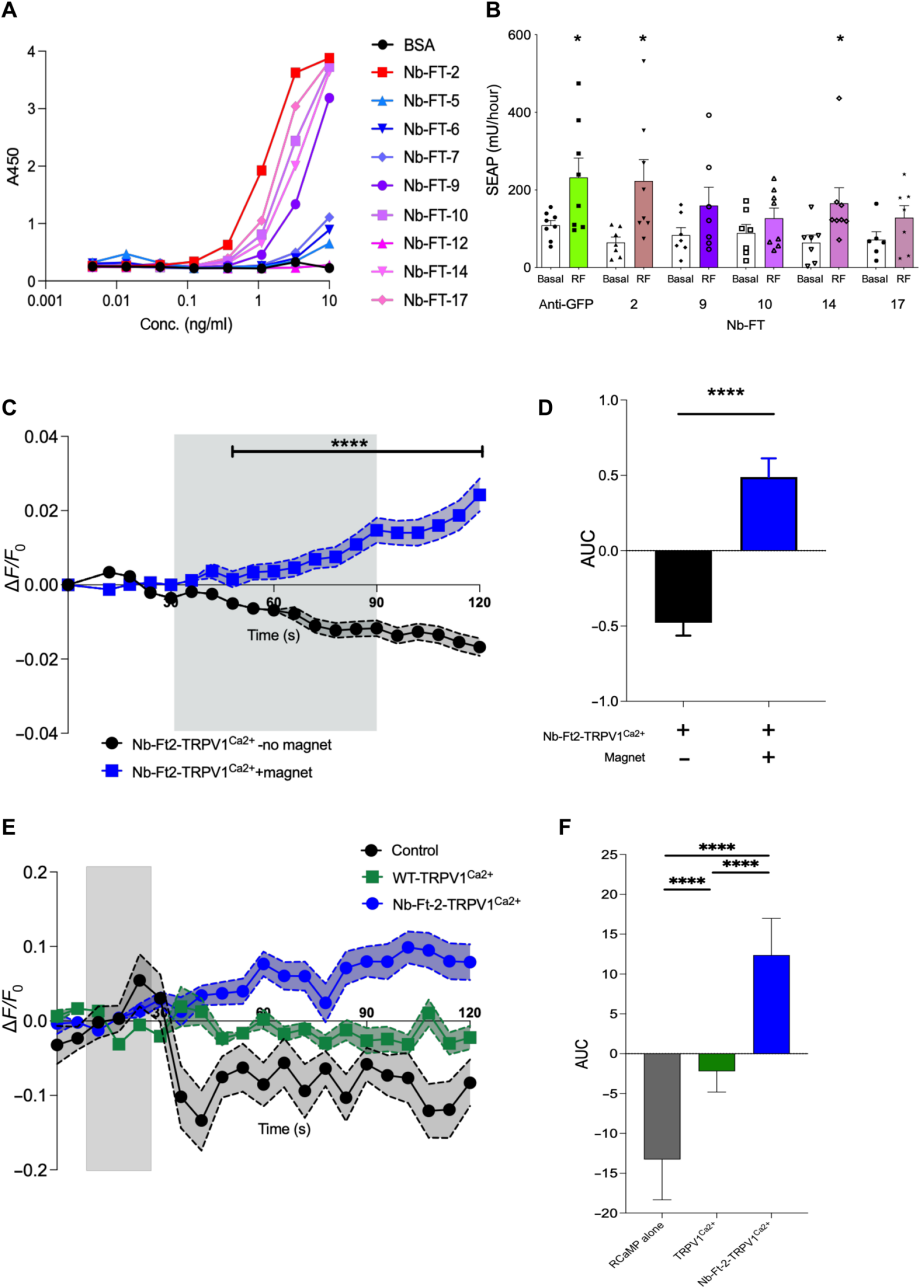
Generation and validation of ferritin-binding nanobodies for magnet stimulation. Serial dilutions of nanobodies or BSA (100 μl) were incubated on plates coated with human spleen ferritin (1 μg/ml) and binding quantified by ELISA after incubation with anti-HA-HRP antibody. (**A**) Quantification of anti-ferritin nanobodies from CDR3 groups 6 and 26 binding to human spleen ferritin. (**B**) Oscillating magnetic field treatment (465 kHz, 30mT) significantly increases calcium-dependent SEAP release from 293T cells transfected with Nb-GFP-TRPV1^Ca2+^, Nb-Ft-2-TRPV1^Ca2+^, and Nb-Ft-14-TRPV1^Ca2+^ and GFP-mFerritin. Data analyzed by two-tailed, unpaired *t* test with Welch’s correction. Nb-GFP-TRPV1^Ca2+^, basal versus RF *P = 0.04, *n* = 8 and 8; Nb-Ft-2-TRPV1^Ca2+^, basal versus RF *P = 0.02, *n* = 7 and 8; Nb-Ft-14-TRPV1^Ca2+^, basal versus RF *P = 0.04, n = 7 and 8. (**C**) Normalized Fluo-4 fluorescence (Δ*F*/*F*_0_) in Neuro2A cells expressing Nb-Ft-2-TRPV1^Ca2+^ with (786 cells) or without (1659 cells) magnet treatment. Data were analyzed by two-way ANOVA with Tukey’s multiple comparison test, ****P < 0.0001. (**D**) Cumulative change in Δ*F*/*F*_0_ of Neuro2A cells expressing Nb-Ft-2-TRPV1^Ca2+^ with (786 cells) or without (1659 cells) magnet treatment. Data were analyzed by Mann Whitney *U* test, ****P < 0.0001. (**E**) Changes in RCaMP fluorescence normalized to baseline fluorescence (Δ*F*/*F*_0_) with magnet treatment of HEK-293T cells expressing RCaMP alone (54 cells), TRPV1^Ca2+^ (107 cells), or Nb-Ft-2-TRPV1^Ca2+^ (101 cells). Error bars represent means ± SEM. (**F**) Cumulative change in Δ*F*/*F*_0_ with magnet treatment of HEK-293T cells expressing RCaMP alone (54 cells), TRPV1^Ca2+^ (107 cells), or Nb-Ft-2-TRPV1^Ca2+^ (101 cells). AUC was calculated for the period of magnet exposure between 48 and 180 s (after focus adjustment) and analyzed by ordinary one-way ANOVA with Tukey’s multiple comparison test, ****P < 0.0001. Data are shown as means ± SD.

Previously, we used a bicistronic construct to deliver a TRPV1–anti-GFP nanobody fusion protein together with green fluorescent protein (GFP)–tagged ferritin (*9*). However, overexpression of exogenously introduced GFP-tagged mouse ferritin could disrupt cellular iron metabolism and this entire system could not be expressed from a single AAV vector. Therefore, we generated endogenous ferritin-binding nanobodies to bind endogenous ferritin and obviated the need for an exogenous ferritin construct. Our screening assay identified 17 ferritin nanobody (Nb-Ft) clones that bound to both human and mouse ferritin (fig. S1, F and G). We selected five Nb-Ft clones with the highest affinity for human ferritin, as assessed by enzyme-linked immunosorbent assay (ELISA) (Fig. 1A), for further study. To test their ability to increase intracellular calcium, we generated constructs with each Nb-Ft clone fused to TRPV1 followed by a 2A peptide cleavage and GFP-tagged mouse ferritin. Oscillating magnetic field treatment (465 kHz, 30 mT) significantly increased calcium-dependent secreted alkaline phosphatase (SEAP) reporter production from human embryonic kidney (HEK) 293T cells transfected with Nb-Ft clones 2 or 14 fused to TRPV1 compared to transfected cells without magnet treatment (basal) (Fig. 1B; Nb-Ft-TRPV1^Ca2+^ clone 2-Basal: 64.7 ± 13.9 mU/hour, Magnet: 223.0 ± 55.0 mU/hour, *P* < 0.05; Nb-Ft-TRPV1^Ca2+^ clone 14-Basal: 64.3 ± 19.4 mU/hour, Magnet: 165.5 ± 19.4 mU/hour, *P* < 0.05).

To evaluate Nb-Ft binding to mouse ferritin, immunoprecipitates (IPs) were analyzed from human HEK-293T cells transfected with Nb-Ft-TRPV1 and GFP-tagged mouse ferritin. IPs from cells transfected with any of the five clones enriched for human endogenous ferritin. However, only IPs from cells expressing clones 2 and 17 were substantially enriched for mouse ferritin compared to expression of GFP-tagged mouse ferritin alone (mouse ferritin enrichment: Nb-Ft clone 2: 14.5-fold enrichment, clone 9: 1.0 clone 10: 1.1, clone 14: 0.9, clone 17: 6.0, and anti-GFP nanobody: 44.3) (fig. S2B and table S1). Together, these data revealed that Nb-Ft clone 2 had the highest affinity for human ferritin, greatest enrichment in mouse ferritin after immunoprecipitation, and significantly increased SEAP release with oscillating magnetic field treatment (fig. S2, C to E). Thus, anti-ferritin nanobody clone 2 (Nb-Ft-2) was selected for further studies.

We next generated an expression vector with Nb-Ft2 fused to TRPV1^Ca2+^ under the control of a neuronal human synapsin promoter (hSyn-Nb-Ft2-TRPV1^Ca2+^), confirmed cell surface channel expression (fig. S3, A and B), and function (fig. S3, C and D). Magnetic field treatment (190 mT) of murine neuro2A cells expressing Nb-Ft2-TRPV1^Ca2+^ significantly increased the mean calcium-dependent Fluo-4 fluorescence [peak Δ*F*/*F*_0_: 0.07 ± 0.005 with magnet versus 0.02 ± 0.001 no magnet, *P* < 0.0001; area under the curve (AUC): 0.49 ± 0.12 with magnet versus −0.47 ± 0.08 no magnet, *P* < 0.0001] (Fig. 1, C and D, and fig. S4A). Similarly, oscillating magnetic field (465 kHz, 30 mT) treatment of human HEK-293T cells expressing Nb-Ft-2-TRPV1^Ca2+^ significantly increased Δ*F*/*F*_0_ compared to cells expressing WT TRPV1^Ca2+^ without Nb-Ft-2 fusion or expressing RCaMP alone (control) (Fig. 1, E and F) and peak Δ*F*/*F*_0_ for each cell [RCaMP fluorescence peak Δ*F*/*F*_0_: 0.10 ± 0.027 Control, 0.11 ± 0.019 WT-TRPV Ca^2+^, 0.28 ± 0.034 Nb-Ft-2-TRPV1^Ca2+^, *P* < 0.0001, Kruskal-Wallis analysis of variance (ANOVA); AUC: −13.27 ± 5.05 Control, −130 2.19 ± 2.61 WT-TRPV1^Ca2+^, 12.38 ± 4.61 Nb-Ft-2-TRPV1^Ca2+^, *P = 0.0195, ****P < 0.0001, 131 Kruskal-Wallis] (fig. S4B). As a positive control, capsaicin treatment of either WT WT-TRPV1^Ca2+^or Nb-Ft-2-TRPV1^Ca2+^ in HEK-293T cells significantly increased RCaMP fluorescence (fig. S3, E and F). These data indicate that magnetic field treatment increases intracellular calcium in both mouse and human cells expressing the single fusion protein Nb-Ft-2-TRPV1^Ca2+^ (referred to hereafter as Nb-Ft-TRPV1^Ca2+^), encoded by a 2.9-kb cDNA, which was used in our AAV vectors for in vivo neuronal transduction.

### Magnetogenetic stimulation of striatal indirect pathway alters motor processing in vivo

**Fig. 2.**
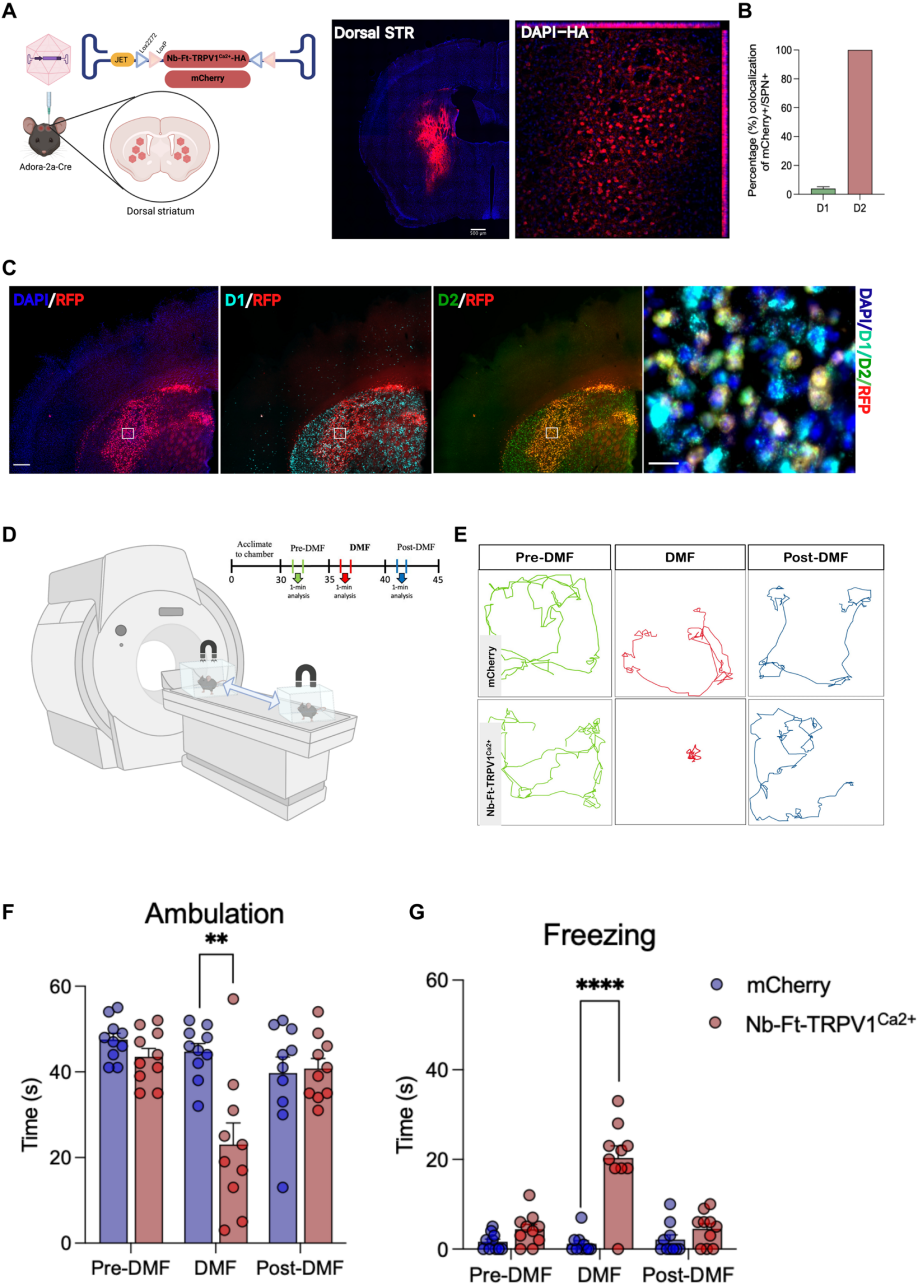
Selective viral-mediated expression of Nb-Ft-TRVP1**^Ca2+^** in striatal iSPNs elicits parkinsonian motor behavior. (**A**) Schema of the double-floxed Cre-dependent AAV vector expressing the Nb-Ft-TRVP1^Ca2+^ or mCherry under the control of the JET promoter; immunostaining for HA in A2a-Cre mice demonstrates selective expression of the AAV- Nb-Ft-TRVP1^Ca2+^ in the dorsal striatum. (**B** and **C**) Selective transduction of D2-type iSPN with RNA in situ hybridization. Colocalization of D2-type (green) iSPN with mCherry (red). (**D**) Protocol and schema for DMF neural activation to assess motor behavior. (**E**) Example of altered motor activity during bilateral striatal pre-DMF (green), DMF (red), and post-DMF (blue) stimulation; individual lines represent the path of a single mouse. Effect of DMF stimulation on (**F**) ambulation bout duration and (**G**) freezing of gait in mCherry (blue bars/dots, *n* = 10) and Nb-Ft-TRVP1^Ca2+^ (red bars/dots, *n* = 10) mice. Error bars show SEM. **P < 0.01, ****P < 0.0001 with two-tailed, unpaired *t* test with Welch’s correction.

For in vivo assessment of functionality of our magnetogenetic construct, we first targeted the striatopallidal circuit also known as the indirect pathway in which activation of dopamine type 2 (D2)–type indirect spiny projection neurons (iSPNs) send GABAergic connections to the globus pallidus externus (GPe), which ultimately results in stimulation of the globus pallidus internus (GPi), inhibition of the thalamus, and therefore inhibition of movement. For this, we used an AAV1/2 vector expressing an hemagglutinin (HA)–tagged, Cre-dependent version of the excitatory magnetogenetic Nb-Ft-TRPV1^Ca2+^ cassette, which was flanked by a double-floxed inverted open reading frame (DIO) under the control of the JET promoter (AAV1/2-JET-DIO-Nb-Ft-TRPV1^Ca2+^-HA). The Cre-dependent Nb-Ft-TRPV1^Ca2+^ AAV was injected bilaterally into the dorsal striatum of transgenic mice expressing Cre recombinase in iSPNs expressing D2 receptors (A2a-Cre mice). Immunostaining for HA or red fluorescent protein (RFP) (for the AAV1/2-JET-DIO-mCherry control group) demonstrated efficient gene expression in the dorsal striatum (Fig. 2A) and projections to the GPe (fig. S5A). Histological analysis of transcript expression using RNAScope confirmed that AAV transcripts colocalized with D2-type neurons and had minimal colocalization with D1-type neurons (D1 = 3.9% versus D2 = 100%, *P* < 0.001) (Fig. 2, B and C); the small percentage of D1 transduction is likely due to double-positive SPN for D1 and D2 RNA probes (fig. S5B). To determine if this new magnetogenetic construct could functionally activate iSPNs in vivo, we first examined motor activity after placing animals in a direct magnetic field (DMF) (Fig. 2D). PD is associated with increased iSPN activity and optogenetic activation of these neurons in normal mice results in rapid freezing (*14*). Bilateral DMF stimulation (500 mT to 1.3 T) using a 3T MRI magnet significantly decreased the activity compared to baseline, with activity returning to normal after removal from the DMF (Fig. 2E). There was also a marked increase in the time spent freezing (*n* = 10, Nb-Ft-TRPV1^Ca2+^ + DMF = 20.3 ± 2.8 versus mCherry + DMF = 1.2 ± 0.69 s, *P* < 0.0001) and a decrease in ambulation (*n* = 10, Nb-Ft-TRPV1^Ca2+^ + DMF = 23.0 ± 5.1 versus mCherry + DMF = 44.7 ± 1.9 s, *P* = 0.001) during DMF application in the Nb-Ft-TRPV1^Ca2+^ group placed in a magnetic field compared to the mCherry group (Fig. 2, F and G). Latency time from DMF exposure until onset of gait freezing was commonly observed between 40 and 60 s following exposure to the magnetic field, and mice returned to motor baseline consistently by 1 min after DMF stimulation was terminated (movie S1). The mCherry group did not show any motor change upon DMF exposure (movie S2). Minor differences in transduction efficiency had no correlation with freezing of gait (fig. S5C).

**Fig. 3.**
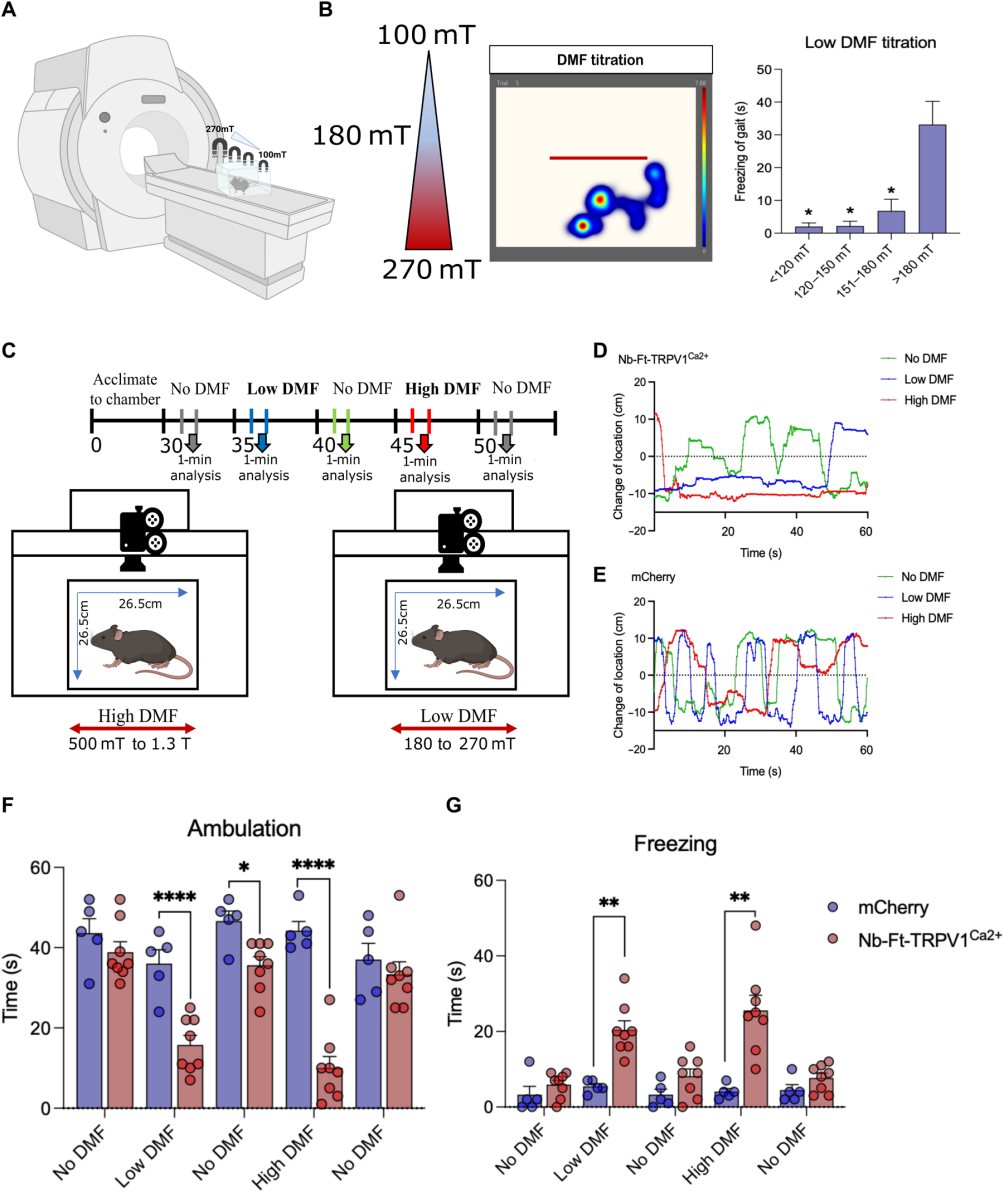
Nb-Ft-TRPV1^Ca2+^ in D2 iSPNs stimulated with high DMF and low DMF treatment produce freezing of gait. (**A**) Schema for low DMF titration. (**B**) Magnetic field strength and individual activity heatmap example of motor activity during bilateral striatal low DMF titration in Nb-Ft-TRPV1^Ca2+^ expressing mice. Freezing of gait in different magnetic field gradients in the low DMF titration ranges (blue bars, *n* = 5) in Nb-Ft-TRPV1^Ca2+^ expressing mice. (**C**) Protocol and schema for high DMF and low DMF stimulation in A2a-Cre mice injected with the double-floxed Cre-dependent AAV vector expressing the Nb-Ft-TRPV1^Ca2+^ or mCherry under the control of the JET promoter. Representative tracking data of change of location in (**D**) Nb-Ft-TRPV1^Ca2+^ and (**E**) mCherry A2a-Cre mice during high DMF (red line), low DMF (blue line), and no DMF (green line) treatment. Effect of high DMF and low DMF stimulation on (**F**) ambulation and (**G**) freezing of gait in Nb-Ft-TRPV1^Ca2+^ (red dot/bars, *n* = 8) and mCherry (blue dots/bars, *n* = 5). Error bars show SEM. *P < 0.05, **P < 0.01, ****P < 0.0001 with two-tailed, unpaired *t* test with Welch’s correction.

To determine the threshold for magnetic field strength required to regulate striatal cellular activity, we compared two different field gradients: 500 mT to 1.3 T (high DMF titration) (fig. S6A) and 100 to 270 mT (low DMF titration), as determined by placing a gaussmeter in different locations in the cage at different distances from the 3T magnet (Fig. 3A). After mapping the magnetic field strength in the behavioral boxes (Fig. 3B and fig. S6B), we then recorded the locomotor activity in animals expressing the magnetogenetic construct. The activity heatmap in the presence of the low DMF revealed a consistent decrease in activity only when the mice receiving Nb-Ft-TRPV1^Ca2+^ were in proximal sections of the behavior box with a field strength at or above 180 mT (Fig. 3B). This contrasted with animals exposed to the high DMF, which showed reduced activity across the entire box (fig. S6C). Thus, while the freezing time was consistent throughout the box in the mice in the high DMF titration (fig. S6D), in the low DMF titration, there was a significant increase in freezing time (*n* = 5, *P* < 0.05) only in the regions with >180-mT field strength (Fig. 3B). This threshold is consistent with a prior report (*10*), which used an earlier generation magnetogenetic system consisting of separate TRPV1 and ferritin cDNAs to demonstrate that a DMF source of ~0.2 T was sufficient to regulate deep brain circuits involved in food intake and glucose homeostasis. To further evaluate if a magnetic field gradient over 180 mT is sufficient to induce a robust motor phenotype, we repeated our behavioral assessment at low DMF and high DMF (Fig. 3C). Consistent with the prior results, we found that mice expressing Nb-Ft-TRPV1^Ca2+^ stayed at a relatively fixed location during both low and high DMF application while control mice expressing mCherry continued to move normally during DMF conditions (Fig. 3, D and E). Indirect pathway activation with both high and low DMF application decreased the time spent ambulating (*n* = 5 to 8, low DMF Nb-Ft-TRPV1^Ca2+^ = 15.75 ± 2.4 versus mCherry = 36 ± 3.48 s, *P* < 0.001; No DMF Nb-Ft-TRPV1^Ca2+^ = 35.63 ± 2.16 versus mCherry = 46.6 ± 2.54 s, *P* < 0.01; high DMF Nb-Ft-TRPV1^Ca2+^ = 10 ± 2.89 versus mCherry = 44.2 ± 2.31 s, *P* < 0.001) and increased time spent freezing (*n* = 5 to 8, low DMF Nb-Ft-TRPV1^Ca2+^ = 20.38 ± 2.4 versus mCherry = 5.4 ± 0.74 s, *P* < 0.01; No DMF Nb-Ft-TRPV1^Ca2+^ = 8.13 ± 1.92 versus mCherry = 3.2 ± 1.46 s, *P* > 0.05; high DMF Nb-Ft-TRPV1^Ca2+^ = 25.5 ± 4.01 versus mCherry = 4 ± 0.83 s, *P* < 0.01) (Fig. 3, F and G).

### Magnetogenetic stimulation increases striatal neuronal activity in vivo

**Fig. 4.**
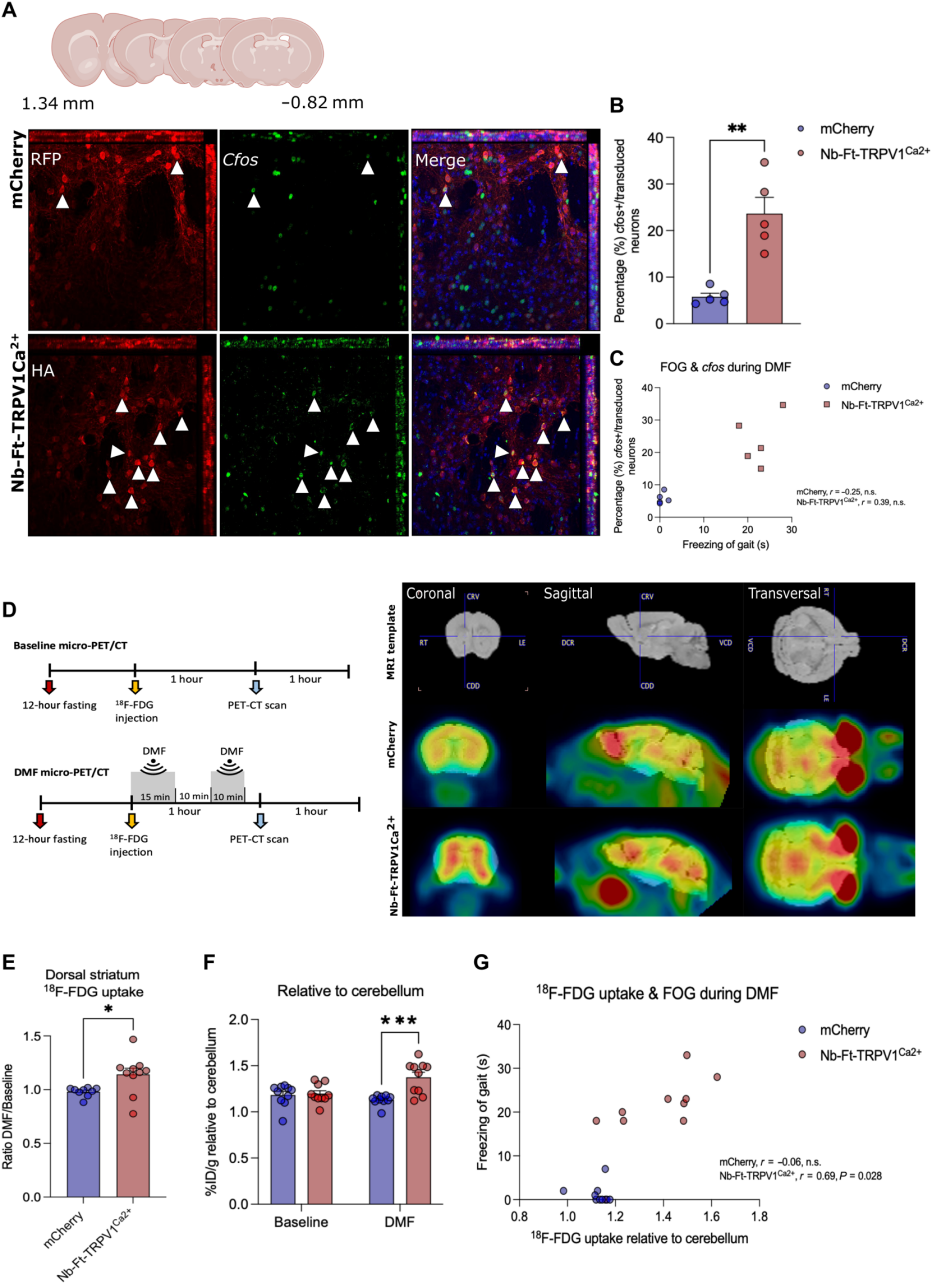
Nb-Ft-TRPV1^Ca2+^ increases *c-fos* expression and cerebral metabolism upon DMF treatment. (**A**) Immunostaining for *c-fos* (cyan) and HA or RFP (red) of the dorsal striatum of A2a-Cre mice euthanized 1 hour following 15 min of DMF exposure. (**B**) Percentage (%) of *c-fos*–positive neurons over total number of iSPNs expressing HA or RFP (*n* = 5 mice per group/3 sections per mice). (**C**) Correlation between *c-fos* expression and freezing of gait during DMF exposure (*n* = 5 mice per group). (**D**) Protocol and schema for ^18^F-FDG micro-PET/CT scan procedure and analysis with representative micro-PET/CT imaging of A2a-Cre mouse brains expressing the Nb-Ft-TRPV1^Ca2+^ or the mCherry control virus following DMF exposure. ^18^F-FDG uptake in the dorsal striatum calculated as the percentage of injected dose/weight (%ID/g). (**E**) DMF over baseline ratio. (**F**) DMF over baseline normalized to the cerebellum, in mCherry (blue bars/dots, *n* = 8) and Nb-Ft-TRPV1^Ca2+^ (red bars/dots, *n* = 8) mice. (**G**) Correlation between DMF over baseline ^18^F-FDG uptake ratio and freezing of gait during DMF exposure (*n* = 10). Error bars show SEM. *P < 0.05, **P < 0.01, ***P < 0.001 with two-tailed, unpaired *t* test with Welch’s correction. Anti-HA staining to detect Nb-Ft-TRVP1^Ca2+^ expression, anti-RFP staining to detect mCherry expression.

We next assessed if the changes in locomotor activity were correlated with changes in neural activity. Previously, the immediate early gene *c-fos* was shown to be increased in vitro and in vivo in cells expressing TRPV1 and ferritin in a magnetic field (*8*, *10*, *11*). Consistent with this, a significantly higher proportion of striatal neurons expressing Nb-Ft-TRPV1^Ca2+^ showed *c-fos*–positive expression compared to mCherry controls following exposure exposed to the MRI magnet (500 mT to 1.3 T) (*n* = 5, Nb-Ft-TRPV1^Ca2+^ = 23.65 ± 3.49 versus mCherry = 5.79 ± 0.77%, *P* < 0.01) (Fig. 4, A and B). There was a correlation between *c-fos* expression and freezing [*n* = 4 to 5, Pearson’s *r* = 0.39, two-tailed not significant (n.s.)] with the Nb-Ft-TRPV1^Ca2+^ and mCherry mice clustering at opposite extremes of the correlation matrix (Fig. 4C). The data are consistent with the result that there is a robust activation of the indirect pathway neurons, with relatively low variability between the expression of gene activity–induced markers and the freezing phenotype.

To further assess striatal activity after magnetogenetic activation living animals, we used PET with [^18^F]fluorodeoxyglucose (^18^F-FDG), which measures glucose utilization as a reflection of underlying neural activity (*17*). Micro-PET/computed tomography (CT) was performed in A2a mice expressing Nb-Ft-TRPV1^Ca2+^ fasted for 12 hours versus mice receiving an mCherry in the striatum construct. ^18^F-FDG was administered to the two groups first without DMF exposure, followed 3 days later by PET imaging following the application of DMF (Fig. 4D, left panel). DMF exposure significantly increased striatal uptake of ^18^F-FDG, expressed as the ratio of uptake at baseline prior to DMF exposure to the magnetic field to the signal after placement in a magnetic field. The ratio of the PET signal [megabecquerel (MBq)/ml] was significantly greater in the Nb-Ft-TRPV1^Ca2+^ group compared to mCherry controls (*n* = 9 to 10, Nb-Ft-TRPV1^Ca2+^ = 1.14 ± 0.06 versus mCherry = 0.98 ± 0.001, *P* < 0.05) (Fig. 4, D, right panel, and E). To account for individual variability, we also quantified the percentage of the ^18^F-FDG dose that was injected dose gram (%ID/g) in the striatum compared to uptake in the cerebellum, with and without DMF treatment. DMF exposure significantly increased ^18^F-FDG uptake in the striatum in mice with Nb-Ft-TRPV1^Ca2+^ expression in D2 neurons relative to mCherry controls (*n* = 10, Nb-Ft-TRPV1^Ca2+^ = 1.37 ± 0.06 versus mCherry = 1.19 ± 0.03 %ID/g, *P* < 0.001) (Fig. 4F). No change in the PET signal was seen in the cerebellum. Because clinically ^18^F-FDG PET-CT is used to monitor PD progression and can be correlated with motor features (*17*), we reasoned that the levels of striatal glucose uptake might correlate with the time mice spent freezing during DMF application. Consistent with this, the data showed a significant positive correlation (*n* = 7 to 9, Pearson’s *r* = 0.69, two-tailed *P* < 0.05) between striatal glucose uptake and freezing time in the Nb-Ft-TRPV1^Ca2+^ expressing mice (Fig. 4G), with the most marked increase in the time spent freezing observed in animals with a ^18^F-FDG uptake over 1.4 using the cerebellum as a reference.

**Fig. 5.**
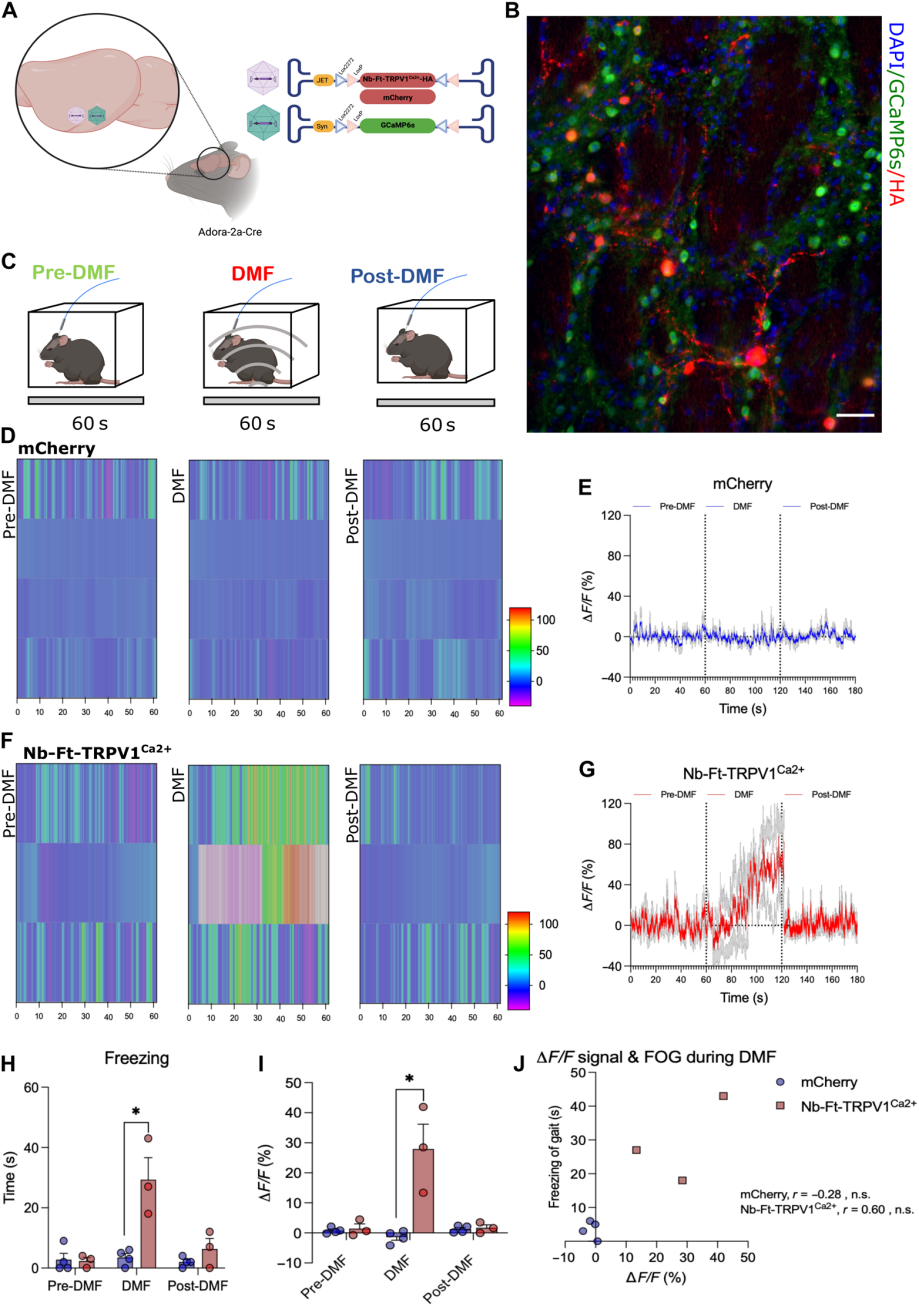
Nb-Ft-TRPV1^Ca2+^ increases calcium transients in the dorsal striatum. (**A** and **B**) Schema and AAV expression of the double-floxed Cre-dependent AAV vector encoding the Nb-Ft-TRPV1^Ca2+^ or mCherry under the control of the JET promoter (red) combined with double-floxed Cre-dependent AAV vector expressing GCaMP6s under the control of the Syn promoter (green). (**C**) Behavioral methodology to assess calcium transients during pre-DMF (green), DMF (red), and post-DMF (blue) treatment. (**F**) Activity trace example of altered motor activity during bilateral striatal pre-DMF (green), DMF (red), and post-DMF (blue) stimulation in an Nb-Ft-TRPV1^Ca2+^ mouse with heatmap of GCaMP fluorescence aligned to pre-DMF (left), DMF (middle), and post-DMF (right) of the same animal shown in activity trace example expressing the Nb-Ft-TRPV1^Ca2+^ vector. (**G**) Mean fluorescence aligned to pre-DMF, DMF, and post-DMF in Nb-Ft-TRPV1^Ca2+^ group (*n* = 3). (**D**) Activity trace example of altered motor activity during bilateral striatal pre-DMF (green), DMF (red), and post-DMF (blue) stimulation in an mCherry mouse with heatmap of GCaMP fluorescence aligned to pre-DMF (left), DMF (middle), and post-DMF (right) for the same animal shown in activity trace example expressing the mCherry vector. (**E**) Mean fluorescence aligned to pre-DMF, DMF, and post-DMF in the mCherry group (*n* = 4). (**H**) Freezing of gait (FOG). (**I**) Average ∆*F*/*F* (%) during pre-DMF, DMF, and post-DMF stimulation. (**J**) Correlation between ∆*F*/*F* (%) calcium transients and FOG during DMF treatment. Error bars show SEM. *P < 0.05 with two-tailed, unpaired *t* test with Welch’s correction.

Last, we used genetically encoded calcium indicators to measure magnetogenetic-induced neural activity (*5*, *7*, *11*, *12*). To selectively record D2 iSPN calcium transients during pre-DMF, DMF, and post-DMF periods, we bilaterally injected a Cre-dependent excitatory magnetogenetic vector (AAV1/2-JET-DIO-Nb-Ft-TRPV1^Ca2+^-HA) together with a Cre-dependent calcium indicator (AAV9-Syn-DIO-GCaMP6s) and implanted an optical fiber in the dorsal striatum of A2a-Cre mice (Fig. 5, A and B). This required the use of optical fibers that did not contain any metal that would heat in the presence of a magnetic field (Fig. 5C). Nb-Ft-TRPV1^Ca2+^ expressing mice again showed motor freezing in response to DMF that was reflected with an increased Δ*F*/*F* striatal GCaMP fluorescence signal (Fig. 5F), which was not seen in mCherry expressing mice (Fig. 5D). Mean GCaMP6 fluorescence (Δ*F*/*F*) percentage aligned to pre-DMF, DMF, and post-DMF revealed a marked increase in fluorescence during DMF stimulation in the Nb-Ft-TRPV1^Ca2+^ group (Fig. 5G) but not in the mCherry group (Fig. 5E). Calcium transients in D2 iSPNs demonstrated by an increase in GCaMP6 fluorescence (Δ*F*/*F*) were significantly increased by DMF treatment in the Nb-Ft-TRPV1^Ca2+^ but not in the mCherry expressing mice (*n* = 3 to 4, Nb-Ft-TRPV1^Ca2+^ + DMF = 27.91 ± 8.24 versus mCherry + DMF = −1.37 ± 1.05%, *P* < 0.05) (Fig. 5I). In parallel, DMF treatment significantly increased time spent freezing in Nb-Ft-TRPV1^Ca2+^ compared to mCherry controls (*n* = 3 to 4, Nb-Ft-TRPV1^Ca2+^ + DMF = 29.33 ± 7.3 versus mCherry + DMF = 3.5 ± 1.3 s, *P* < 0.05) (Fig. 5H), confirming the functional efficacy of magnetogenetic activation. Consistent with the *c-fos* expression level and the PET studies, there was a positive correlation between the change in fluorescence signal and the extent of the motor inhibition (*n* = 3 to 4, Pearson’s *r* = 0.60, two-tailed n.s.), with the experimental and control groups mostly clustering at opposite ends of the correlation matrix (Fig. 5J). This combined data demonstrate that the fused ferritin nanobody-TRPV1 channel, expressed from a single AAV in striatal D2 neurons, resulted in activation of transduced iSPNs, which then caused the resulting motor freezing.

### TMS increases Nb-Ft-TRPV1^Ca2+^–mediated calcium transients and elicits parkinsonian phenotype

**Fig. 6.**
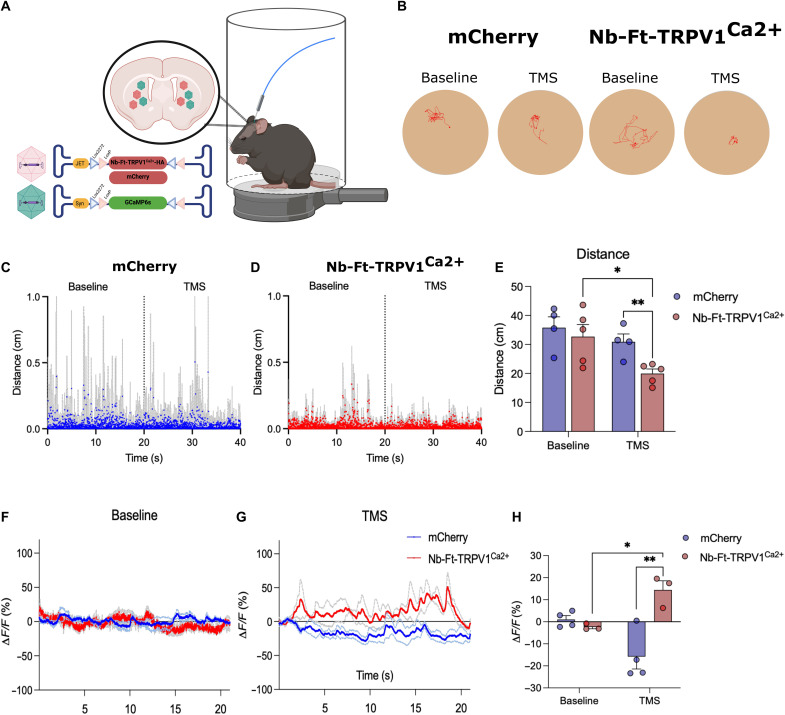
TMS treatment increases Nb-Ft-TRPV1^Ca2+^–mediated motor freezing and calcium transients in the dorsal striatum. (**A**) Schema of the TMS setup and A2a-Cre mice with bilateral injection of the double-floxed Cre-dependent AAV vector expressing the Nb-Ft-TRPV1^Ca2+^ or mCherry under the control of the JET promoter (red) combined with double-floxed Cre-dependent AAV vector expressing GCaMP6s under the control of the Syn promoter (green) in the dorsal striatum. (**B**) Activity trace example of altered motor activity during baseline (left) and TMS (right) treatment in an mCherry mouse (blue) and Nb-Ft-TRPV1^Ca2+^ mouse (red). Distance ambulated during baseline (left) and TMS (right) treatment in the (**C**) mCherry group (blue) and (**D**) Nb-Ft-TRPV1^Ca2+^ group (red) (*n* = 4 to 5; data presented as the means ± SEM in gray). (**E**) Average distance during baseline and TMS treatment in mCherry (blue) and Nb-Ft-TRPV1^Ca2+^ groups. (**F**) Mean fluorescence during baseline in Nb-Ft-TRPV1^Ca2+^ (red) and mCherry (blue) mice (*n* = 3; data presented as the means ± SEM). (**G**) Mean fluorescence during 20 s of TMS triggers every 2 s in Nb-Ft-TRPV1^Ca2+^ (red) and mCherry (blue) mice (*n* = 3 to 4; data presented as the means ± SEM). (**H**) Grouped bar graph of Nb-Ft-TRPV1^Ca2+^ (red) and mCherry (blue) mice during baseline and 20 s of TMS triggers every 2 s (*n* = 3 to 4). Error bars show SEM. *P < 0.05, **P < 0.01 with two-tailed, unpaired (or paired when baseline versus TMS comparison were done) *t* test with Welch’s correction.

While the MRI magnet was highly effective for demonstrating and characterizing the magnetogenetic effect of the new construct on neurons within the brain, the feasibility of translating into human application a technology that requires proximity to an MRI for therapeutic efficacy is limited. To evaluate the potential of a more clinically relevant technology, we repeated the studies above using a commercial TMS system, which is Food and Drug Administration approved for depression and other applications (Fig. 6A) (*18*). Similar to the effect of an MRI machine, TMS application at 20% output capacity using 50 twin pulses per second with 1-s interstimulus intervals decreased motor ambulation in Nb-Ft-TRPV1^Ca2+^ expressing mice compared with mCherry controls (Fig. 6B). Analysis of motor parameters including distance moved (Fig. 6, C and D), activity (fig. S7, A and B), and change of location (fig. S7, D and E) was consistent with activation of D2 iSPN in Nb-Ft-TRPV1^Ca2+^ expressing mice compared to mCherry controls. Average distance moved and activity percentage during TMS application was significantly decreased in the Nb-Ft-TRPV1^Ca2+^ expressing mice from baseline (*n* = 5, Nb-Ft-TRPV1^Ca2+^ + Baseline = 32.75 ± 4.15 versus Nb-Ft-TRPV1^Ca2+^ + TMS = 19.98 ± 1.57 cm, *P* < 0.05) and compared to mCherry controls (*n* = 4 to 5, Nb-Ft-TRPV1^Ca2+^ + TMS = 19.98 ± 1.57 versus mCherry + TMS = 30.94 ± 2.71 cm, *P* < 0.01) (Fig. 6E and fig. S7C). Again, using targeted GCAMP expression and fiber photometry, TMS treatment produced rapid generation of fluorescent positive spikes in the striatum of Nb-Ft-TRPV1^Ca2+^ expressing mice while TMS treatment showed a sustained decrease in GCaMP6 fluorescence in mCherry-expressing controls (Fig. 6, F and G) (*19*). Fluorescence (Δ*F*/*F*) quantification showed a significant increase in the Nb-Ft-TRPV1^Ca2+^ expressing mice compared to their own baseline (*n* = 3, Nb-Ft-TRPV1^Ca2+^ + TMS = 14.41 ± 4.15 versus Nb-Ft-TRPV1^Ca2+^ + Baseline = −2.38 ± 0.81%, *P* < 0.05) and compared with mCherry mice during TMS application (*n* = 3 to 4, Nb-Ft-TRPV1^Ca2+^ + TMS = 14.41 ± 4.15 versus mCherry + TMS = −15.81 ± 5.61%, *P* < 0.01) (Fig. 6H). These studies confirm that, as was seen with MRI-generated magnetic fields, a clinically approved TMS device is capable of activating transduced neurons and eliciting motor freezing in Nb-Ft-TRPV1^Ca2+^ expressing mice at well below the maximal output of the device.

### Magnetogenetic stimulation of striatopallidal neurons in WT mice reproduces a robust motor phenotype

**Fig. 7.**
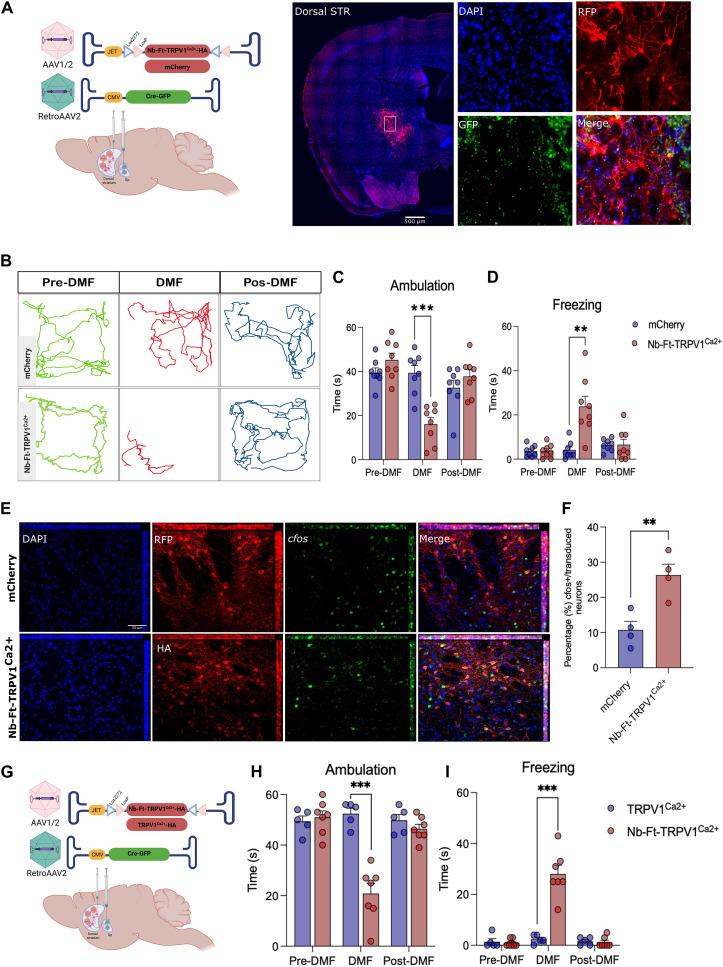
Selective viral-mediated expression of Nb-Ft-TRPV1^Ca2+^ in the striatopallidal pathway elicits parkinsonian motor behavior in WT mice. (**A**) Schema of the double-floxed Cre-dependent AAV vector expressing the Nb-Ft-TRPV1^Ca2+^ or mCherry under the control of the JET promoter in striatal iSPN projecting to the Gp achieved by Gp injection of a retrograde AAV2 vector carrying Cre-GFP; immunostaining for GFP (green) and HA (red) in WT mice demonstrates selective expression of the AAV- Nb-Ft-TRPV1^Ca2+^ in the dorsal striatum iSPN projecting to the GPe; white arrows show colocalization of GFP with HA. (**B**) Example of altered motor activity during bilateral striatopallidal pre-DMF (green), DMF (red), and post-DMF (blue) stimulation; lines represent the mouse’s path. Effect of DMF stimulation on (**C**) ambulation bout duration and (**D**) freezing of gait in mCherry (blue bars/dots, *n* = 8) and Nb-Ft-TRPV1^Ca2+^ (red bars/dots, *n* = 8) mice. (**E**) Immunostaining for *c-fos* (cyan) and HA (Nb-Ft-TRVP1^Ca2+^; red) or RFP (mCherry; red) of the dorsal striatum of WT mice euthanized 1 hour following 15 min of DMF exposure. White arrows show colocalization. (**F**) Percentage (%) of *c-fos*–positive neurons over the total number of iSPNs expressing HA or RFP (*n* = 3 mice per group/3 sections per mice). (**G**) Schema of the double-floxed Cre-dependent AAV vector expressing the Nb-Ft-TRPV1^Ca2+^ or TRPV1^Ca2+^ under the control of the JET promoter in striatal iSPNs projecting to the Gp achieved by Gp injection of a retrograde AAV2 vector carrying Cre-GFP. Effect of DMF stimulation on (**H**) ambulation bout duration and (**I**) freezing of gait in TRPV1^Ca2+^ (blue bars/dots, *n* = 5) and Nb-Ft-TRPV1^Ca2+^ (red bars/dots, *n* = 7) mice. Error bars show SEM. **P < 0.01, ***P < 0.001 with two-tailed, unpaired *t* test with Welch’s correction.

Cre-driver mouse lines provide cell specificity for functional mapping of neural circuits underlying numerous behaviors and many diseases, but this approach is limited to animals. In addition, expression is based on cellular identity and not on functional anatomical connections (*20*). To determine if magnetogenetics could be restricted to specific circuits in normal mice, we used a dual vector system to restrict expression to the striatopallidal iSPN circuitry. AAV1/2-JET-DIO-Nb-Ft-TRPV1^Ca2+^-HA was first injected bilaterally into the dorsal striatum of normal mice, which would not lead to expression of the viral transgene due to the absence of Cre. This was followed by a second injection of a retrograde AAV vector carrying a Cre recombinase fused to GFP (RetroAAV2-CMV-Cre-GFP) into the globus pallidus (Gp). This approach allows retrograde uptake of AAV into afferents to the Gp, with resulting Cre expression in neurons that project to Gp, including D2 iSPNs. Because the only pallidal afferents containing a floxed transgene with this approach would be those from the dorsal striatum, this should lead to restricted expression of the magnetogenetic construct only in striatopallidal neurons. As expected, immunostaining for HA or RFP (for AAV1/2-JET-DIO and the mCherry control group, respectively) demonstrated colocalization of each with GFP from the transduced SPNs (Fig. 7A). The same behavioral tests were then performed as were used to previously characterize the transgenic A2a-Cre mice (Fig. 2). Consistent with the prior results, there was a robust decrease in locomotor activity when the animals receiving the AAV1/2-JET-DIO-Nb-Ft-TRPV1^Ca2+^-HA injections were placed in a magnetic field compared to controls (Fig. 7B). There was a significant decrease in ambulation during DMF application (*n* = 8, Nb-Ft-TRPV1^Ca2+^ + DMF = 16.13 ± 3.02 versus mCherry + DMF = 39.38 ± 3.28 s, *P* < 0.001) and a significant increase in time spent freezing (*n* = 8, Nb-Ft-TRPV1^Ca2+^ + DMF = 23.75 ± 4.63 versus mCherry + DMF = 4.13 ± 1.34 s, *P* < 0.001) (Fig. 7, C and D). *c-fos* expression was again significantly higher in the transduced iSPNs of Nb-Ft-TRPV1^Ca2+^ expressing mice compared to mCherry controls (*n* = 4, Nb-Ft-TRPV1^Ca2+^ + DMF = 26.34 ± 3.13 versus mCherry + DMF = 10.76 ± 2.41%, *P* < 0.01) (Fig. 7, E and F). To control for the possibility that striatal overexpression of TRPV1 ion channels alone could mediate activation of the indirect pathway in the absence of ferritin tethered to the TRPV1 channel, we compared the effects of DMF treatment on WT mice expressing either Nb-Ft-TRPV1^Ca2+^ or TRPV1^Ca2+^ alone (Fig. 7G). DMF application had no observable motor effect on TRPV1^Ca2+^ expressing mice but again suppressed ambulation and increased freezing in mice expressing Nb-Ft-TRPV1^Ca2+^, confirming that fusion of the ferritin nanobody to the TRPV1 channel was required for neuronal activation in the presence of a magnetic field (Fig. 7, H and I).

### Magnetogenetic inhibition of STN neurons improves motor impairment in parkinsonian mice

**Fig. 8.**
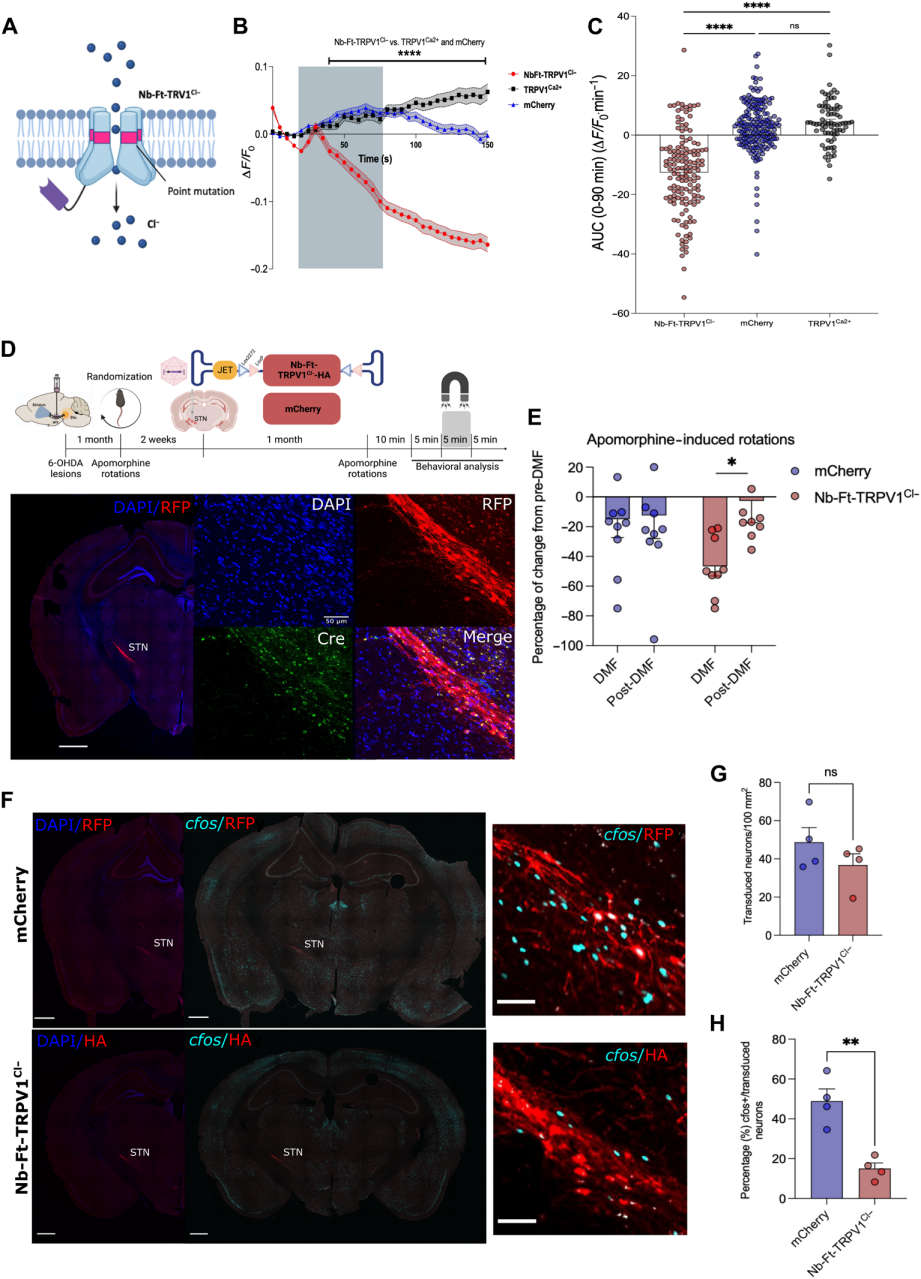
Mutant Nb-Ft-TRPV1^Cl−^ inhibits neuronal activity of STN projection neurons and rescues motor impairment in parkinsonian mice. (**A**) Schema of inhibition system with mutant Nb-Ft-TRPV1^Cl−^ membrane channel. (**B**) Normalized change in MQAE fluorescence intensity (Δ*F*/*F*_0_) of Neuro2A cells expressing mCherry, TRPV1^Ca2+^, or Nb-Ft-TRPV1^Cl−^ with low AMF application in vitro. (**C**) AUC analysis of chloride imaging for the Nb-Ft-TRPV1^Cl−^ channel. (**D**) Protocol and schema for generation of parkinsonian PitX2-Cre mice with unilateral injection of 6-OHDA in the MFB, followed by randomization-based contralateral rotations induced by intraperitoneal administration of apomorphine (0.25 mg/kg) and ipsilateral intracranial injection of the double-floxed Cre-dependent AAV vector expressing the mutant Nb-Ft-TRPV1^Cl−^ under the control of the JET promoter in the STN. Representative immunostaining of the selective expression of Nb-Ft-TRPV1^Cl−^ (red) in STN neurons expressing Cre recombinase (green). (**E**) Percentage of change from pre-DMF treatment in contralateral rotations induced with apomorphine (0.25 mg/kg, ip) in parkinsonian PitX2-Cre mice expressing the Nb-Ft-TRPV1^Cl−^ or the mCherry control viruses in the STN during DMF and post-DMF. (**F**) Immunostaining for *c-fos* (cyan) and HA or mCherry (red) of STN of PiX-2-Cre mice euthanized 1 hour following 15 min of DMF exposure with apomorphine-induced rotations (0.25 mg/kg). (**G**) Number of transduced neurons per area in STN between mCherry and Nb-Ft-TRPV1^Ca2+^ groups. (**H**) Percentage (%) of *c-fos*–positive neurons over the total number of STN neurons expressing HA or RFP (*n* = 4 mice per group/3 sections per mice). Error bars show SEM. *P < 0.05, **P < 0.01 with two-tailed, unpaired *t* test with Welch’s correction. Data were analyzed by Mann Whitney U test, ****P < 0.001. Anti-HA staining to detect Nb-Ft-TRVP1^Ca2+^ expression, anti-RFP staining to detect mCherry expression.

One advantage of chemogenetic and optogenetic systems is the availability of a suite of modified channels that can inhibit neurons in addition to those that can activate neuronal firing (*21*). Developing an inhibitory magnetogenetic system would not only expand the opportunities for scientific research using this technology but would also provide translational opportunities for diseases where neuronal inhibition would be desirable. We first validated in vitro the utility of the anti-ferritin nanobody fused to a mutant TRPV1 channel modified to allow permeability of chloride (Cl^−^) ions (Nb-Ft-TRPV1^Cl−^), which should increase chloride flux to inhibit neuronal activity (Fig. 8A). Neuro2A cells were transfected with plasmids expressing mCherry, TRPV1^Ca2+^, or Nb-Ft-TRPV1^Cl−^ for fluorescent chloride imaging using N-[ethoxycarbonylmethyl]-6-methoxy-quinolinium bromide (MQAE), which is quenched by intracellular chloride (fig. S8A). Application of a low-intensity magnetic field (230 to 250 mT) showed a significant decrease in the Δ*F*/*F*_0_ in the Nb-Ft-TRPV1^Cl−^ expressing cells compared to both mCherry and TRPV1^Ca2+^ controls (Δ*F*/*F*_0_ at 150 s: Nb-Ft-TRPV^Cl−^ −0.16 ± 0.01, TRPV1^Ca2+^ 0.06 ± 0.01, mCherry −0.002 ± 0.01, *P* < 0.001) (Fig. 8, B and C), consistent with increased intracellular chloride concentrations.

The use of an inhibitory magnetogenetic system may be of potential use for symptomatic treatment of PD, where chemical or gene therapy–based inhibition of STN projection neurons can improve motor function in rodents, nonhuman primates, and humans (*22*–*26*). To test this, we generated unilateral 6-hydroxydopamine (6-OHDA) lesions of nigral dopaminergic neurons in transgenic mice expressing the Cre recombinase in PitX2-positive neurons (PitX2-Cre mice) (fig. S8B), which restricts Cre expression to STN glutamatergic projection neurons with virtually no expression within other populations in the vicinity (*27*). Stable and functionally significant lesions were documented through quantification of apomorphine-induced rotations, followed by randomization of animals to balanced experimental and control groups (fig. S8C). Before AAV injections, another session of apomorphine-induced rotations was done inside the MRI to confirm that DMF alone does not affect rotations and to select the window of time in which rotations remain stable for the STN inhibition experiment (fig. S8D). A DIO Cre-dependent AAV1/2 vector encoding the Nb-Ft-TRPV1^Cl−^ cassette under the control of the JET promoter (AAV1/2-JET-DIO- Nb-Ft-TRPV1^Cl−^-HA) was then injected into the ipsilateral STN of 6-OHDA–lesioned PitX2 mice, with another group injected with a DIO Cre-dependent mCherry vector as a negative control (Fig. 8D). Mice expressing Nb-Ft-TRPV1^Cl−^ in the STN showed a significant reduction in contralateral rotations induced by the dopaminergic agonist, apomorphine, in the presence of a DMF application relative to baseline and control baseline (*n* = 9 to 10, −46.62 ± 6.5 versus −2.67 ± 14.69%, *P* < 0.05) (Fig. 8E, fig. S8, E and F, and table S2). The number of *c-fos*–positive transduced neurons in the STN of PitX2-Cre mice was significantly decreased following DMF exposure in the group that received the inhibitory magnetogenetic Nb-Ft-TRPV1^Cl−^ vector compared to mCherry controls (*n* = 4, Nb-Ft-TRPV1^Cl−^ = 15.01 ± 2.83 versus mCherry = 48.89 ± 6.11%, *P* < 0.01) (Fig. 8, F and G). This confirms that AAV-mediated expression of the magnetogenetic inhibitory construct exclusively within PitX2-positive STN projection neurons resulted in reduction in neuronal activity in vivo and a consequent improvement in motor function following 6-OHDA lesioning of the substantia nigra.

## DISCUSSION

The demonstration that viral vectors such as AAV can be used for gene transfer in the mammalian brain (*28*) has transformed preclinical neuroscience research. Furthermore, many completed and active clinical trials of gene therapy in humans have shown promise for treating circuit disorders (*26*, *29*). While, initially, genes cloned into AAV were expressed constitutively, more recently techniques such as optogenetics and chemogenetics have been used to regulate neural activity in an inducible manner, using light or drugs, respectively. Optogenetics provides temporally rapid and precise control of neuronal function but uses microbial-derived channels that can be immunogenic and require permanent implantation of light-generating devices that potentially tether small animals and present engineering and safety challenges for human applications (*4*, *30*). Chemogenetics uses drugs to regulate channels, which obviates the need for a device implant, but chemogenetic regulation occurs in a more delayed manner based on the pharmacokinetics of the activator molecules (*31*, *32*). Here, we validate magnetogenetics as an alternative approach for modulating neural activity and show that magnetic field–based regulation of neuronal function can be used for bidirectional control of neuronal activity. This method provides both anatomical and temporal precision in awake, freely moving animals without the need for an implanted device and uses a viral vector potentially applicable for clinical studies and can respond to clinically approved devices, which deliver sufficient strength magnetic fields. While the activation of Nb-Ft-TRPV1^Ca2+^ transduced neurons in our experiments did not allow for millisecond-timescale control of neuronal spiking offered by techniques such as optogenetics, the onset of phenotypes was seen in the order of seconds following DMF stimulation, which significantly improves the temporal resolution achieved by chemogenetic-based methods while eliminating the need for implants required for optogenetics. The reproducibility of timing of behavioral responses to magnetic fields across experiments also supports the predictability of this approach.

We created this system using variants of TRPV1, which are endogenous mammalian channels, together with a nanobody that binds to endogenous mouse and human ferritin (*33*). TRPV1 is attractive as a channel as it is less sensitive to temperature and osmotic changes than some other TRP channels such as TRPV4, which may reduce nonspecific activation of the channel itself in the absence of a magnetic field (*34*). We tested this approach by eliciting a parkinsonian phenotype in freely moving mice to demonstrate the functional efficacy of magnetogenetic activation of striatal indirect medium spinal neurons. Activation of these neurons in a magnetic field was evident only in mice receiving the magnetogenetic construct compared with controls, as confirmed by *c-fos* expression post-mortem as well as both FDG-PET and fiber photometry in living animals. Optogenetic control of basal ganglia circuitry has previously demonstrated similar changes in motor behavior after selective activation of indirect pathways in vivo using an implanted light device to control neurons expressing Channelrhodopsin-2 (ChR2) (*14*). More recently, Munshi *et al.* (*5*) reported successful magnetogenetic modulation of motor circuits targeting the motor cortex and dorsal striatum. Consistent with the classical model of basal ganglia circuitry, this study reported a decrease in voluntary movement by activation of striatal D2 iSPN using a combination of metal nanoparticles (MNPs) and AAVs encoding TRPV1 channels stereotactically injected 4 weeks apart in the ridge between the dorsal and ventral striatum (*5*). However, synthetic magnetic nanoparticles would not allow for stable, chronic magnetogenetic activation and the use of MNPs could potentially tether to other thermosensitive channels causing off-target activation and safety issues (*7*, *35*). In addition, the intrinsic variability of separate injections and non–cell-specific transgene transduction can limit conclusions regarding the relationship between phenotype and cells modulated by the exogenous particle–based magnetogenetic system. This is in contrast to the genetically encoded system that we used, which only requires injection of a single AAV to improve both dosing consistency between individuals and longevity of neuromodulation due to chronic AAV-mediated expression. For human translation, the ability to deliver a single construct with a single infusion has both safety and regulatory advantages, and here we demonstrate the viability of AAV-mediated delivery of a single magnetogenetic construct encoding a fused Nb capable of tethering endogenous ferritin to an excitatory TRPV1 channel for inducible modulation of neuronal activity leading to definable motor phenotypes. In this study, there was no evidence of toxicity from either the construct or activation of the Nb by a magnetic field, as evidenced by the repeated behavioral responses over several months upon exposure to the magnetic field with return to baseline each time, as well as histological evidence of equivalent transduction and striatal D2 neurons at the end of months of repeated testing compared with controls. Nevertheless, future preclinical studies should explore if binding endogenous ferritin to the Nb-Ft-TRPV1^Ca2+^ channel could substantially reduce cellular ferritin pools and disrupt normal iron metabolism in neurons with consequences in cell health and the overall safety of the procedure and also examine different lengths and patterns of magnetic field exposure to determine if channel activation can be toxic under circumstances other than those tested here.

The incorporation of optogenetics in neuronal circuit studies, through a combination of focal delivery of viral constructs expressing opsins and implantation of light emitting devices in a target brain region, has led to a wealth of knowledge regarding the relevance of neuronal circuits to particular behaviors (*36*). The potential benefit of the magnetogenetic system described here is the use of a noninvasive magnetic field to regulate specific circuits in freely moving animals, rather than a permanent optical fiber implant that is tethered to an external device. We first demonstrated that a Cre-dependent magnetogenetic system can target particular neuronal populations in Cre-driver mice. However, this approach is limited to animals and may also not provide the anatomical specificity required to examine anatomical circuits. To address this, we also developed a modified approach combining delivery of the Cre-dependent magnetogenetic activating construct into the striatum with injection of a retrograde viral vector (AAVretro) into the Gp, a known target of striatal iSPNs, to restrict expression of the activating construct exclusively within the same striatopallidal iSPNs targeted in the A2a-Cre driver mice. Histological analysis confirmed that expression was restricted to striatal D2 neurons, and the behavioral and physiological responses to the magnetic field were similarly robust compared with data generated in the A2a-Cre mice. However, AAVretro may not efficiently transduce all neurons in a retrograde manner and must be individually tested for particular applications, although there are alternative retrograde viral vectors that could also be used in such an approach if the pattern of retrograde uptake differs between vectors (*37*). Nonetheless, our data provide a novel means for noninvasive regulation of specific neuronal circuits without the need for transgenic animals, which could not only be useful for preclinical research but even have the potential for circuit-specific human translation.

One possible limitation of the application of magnetogenetics in a clinical setting is the availability of a device for generating a magnetic field that is suitable for human therapeutic applications. Stanley *et al.* (*10*) previously reported successful bidirectional magnetogenetic modulation of ventromedial hypothalamus neurons using the earlier dual construct system and an MRI to deliver the magnetic field, and we similarly observed a robust motor phenotype following exposure to a 3T MRI in mice expressing Nb-Ft-TRPV1^Ca2+^ in iSPNs at similar field strength thresholds. While this is feasible for experimental studies, any requirement of proximity to a 3T MRI for efficacy could limit the translational potential of this technology. We addressed this by also testing a TMS device as a source of AMF to induce magnetic field–based stimulation of D2 iSPNs. As with the MRI machine, we observed a sustained effect from the TMS-generated magnetic field in mice expressing Nb-Ft-TRPV1^Ca2+^ in striatal D2 iSPNs, with decreased locomotion and corresponding increase in GCaMP fluorescence. In vivo fiber photometry analysis also revealed that TMS reduced neural activity in control mice, which could potentially be attributed to TMS frequency-dependent neural activation-inhibition. This is not unexpected as TMS does influence neuronal activity in isolation, which is the basis for current clinical applications. Human (*38*) and animal (*19*) reports have shown suppression of neural activity in motor-related regions with different protocols of TMS treatment, especially low-frequency TMS. Nonetheless, this reduced activity had no behavioral effect and if anything suggests that we may even have underestimated the magnitude of magnetogenetic neuronal activation. Given the widespread and increasing use of TMS as a clinical device for noninvasive neuromodulation in a variety of conditions (*18*), it is possible that magnetogenetics could be used to either augment TMS or allow TMS to target deeper neuronal populations where the strength of the magnetic field weakens but is still above the threshold that we have observed for functional activation of the recombinant engineered channel. Field mapping of the TMS device used in this study (see Materials and Methods) allow us to estimate that, at 100% capacity, a 3T butterfly coil could reach between 2 to 3 kT/s in brain nuclei at −50 to −70 mm beneath the surface of the skull, which is sufficient distance to reach most deep brain targets above the brainstem that are relevant to neurological and psychiatric disease. In a biphasic pulse mode of ~280 ms, the field strength would result in ~0.3 T, a field gradient sufficient to induce magnetogenetics-dependent modulation but insufficient to produce a hypothetical stochastic effect in nontransduced neurons that receive this magnetic flux. Predictions of the magnetic field gradients that could safely reach deep brain structures should be considered carefully in the context of several biological and physical aspects such as the TMS-induced currents that potentially modify the field deep within a skull, the TMS properties (coil, wiring, and capacity), and characteristic of generated pulses (width and waveform). Nonetheless, the possibility to generate low–field strength at-home devices for intermittent modulation similar to TMS, while maintaining anatomical specificity through focal delivery of the gene therapeutic, could also allow broader application of this technology. Last, the ability to regulate peripheral or autonomic neurons is plausible using this technology for indications where either activation or inhibition of neural activity is desirable, potentially including the control of pain, neuropathy symptoms, or organ function. Our data establishing the threshold for magnetic field strength also raise the possibility that other more simple or even wearable magnetic devices may enable regulation of more accessible neuronal populations. The current study establishes the viability and basic parameters of the magnetogenetic system and raises a variety of possibilities for translation, but considerable future development and characterization would be needed for specific applications that could have unique requirements and challenges.

To expand the utility of this system, we have also developed a novel construct with a mutation that converted the sodium channel into a chloride channel, allowing magnetogenetic inhibition of neuronal activity. Our in vitro data demonstrated the functionality of this new construct, while AAV-mediated delivery of the construct to a population of glutamatergic projection neurons within the STN of hemiparkinsonian PitX2-Cre mice confirmed the in vivo efficacy of magnetogenetic inhibition and consequent motor improvements in this setting. PitX2 expression is restricted to neurons within the general region of the STN (*39*, *40*), allowing very limited expression of our Cre-conditional magnetogenetic system to this population of STN neurons. Altered activity of the STN is widely believed to be one of the pathophysiological mechanisms leading to PD motor deficits (*41*); hence, traditional high-frequency DBS in the STN has emerged as a standard therapy for advance stages of PD (*2*). Nonetheless, there is equipoise regarding the mechanism of DBS action and the role of inhibition of STN neurons as a PD therapeutic. Gradinaru *et al.* (*41*) found that optical inhibition of glutamatergic STN neurons in hemiparkinsonian rats was insufficient to affect motor behavior using early generation inhibitory opsins, yet STN lesioning, infusion of γ-aminobutyric acid (GABA) agonists into the STN, and gene therapy to promote GABA production within the STN have all demonstrated improved motor function in both preclinical studies and human clinical trials (*22*–*24*, *26*, *42*). While DBS may still function through mechanisms other than simple neuronal inhibition (*42*), our results support a body of evidence suggesting that inhibition of STN projection neurons can improve motor function in PD (*22*, *26*, *44*–*47*). The availability of an inhibitory magnetogenetic system, which can also be combined with retrograde Cre vectors to target specific populations and can be applied to peripheral neurons as well, could vastly expand both the experimental and clinical potential of this technology. We acknowledge, however, that a potential limitation for therapeutic applications of magnetogenetics is the ubiquity of magnetic field–based imaging as an important diagnostic tool for human diagnostics, which could influence cells expressing magnetogenetic channels. While there are devices currently in human use that preclude MRI imaging, so this is accepted in human practice, further development of magnetogenetic technology likely could provide solutions to avoid this problem. For example, temporal regulation of gene expression through synthetic biology developments, such as ribozymes/riboswitches fused to aptamers, which could block expression in the presence of an inducer agent, could allow for selective time windows to decrease translation of the magnetogenetics channels when magnetic field interventions are planned.

The data reported here firmly establish that expression of a TRPV1 channel tethered to ferritin in vivo regulates neuronal activity in the presence of a magnetic field using several different independent readouts, including real-time calcium signaling, PET-based glucose utilization and immediate early gene expression. Together with the corresponding behavioral changes, these data confirm that the TRPV1-ferritin fusion proteins that we developed can bidirectionally modulate neural activity in vivo in response to a magnetic field of sufficient strength. While other groups have also published empiric data confirming a cellular response with magnetogenetic activating constructs using different readouts (*7*, *48*), the possibility that mechanical or thermal gating as mechanisms of regulating the channel was initially questioned on purely theoretical grounds (*49*). However, more recently several mechanisms have been proposed to explain gating of TRP channels tethered to ferritin in a magnetic field. Detailed theoretical analyses of a range of potential physical parameters of ferritin-iron nanoparticles and their interaction with the mechanosensitive ion channel suggest the possibility that alternative physical forces can trigger channel opening (*51*). These include possible magneto-caloric and magneto-thermal mechanisms, with another study raising the possibility that a magneto-caloric mechanism induces a thermally mediated magnetic response via magnetic entropy (*52*). In addition, several groups have generated data to support a possible chemical mechanism, in which exposure of ferritin to either a magnetic or radiofrequency field increases the cellular levels of reactive oxygen species with lipid oxidation changing TRP channel dynamics (*35*, *53*, *54*). These possibilities are not mutually exclusive, and both chemical and physical factors may contribute. Given that the mentioned physical and chemical mechanisms could result in distinct TRPV1 gating time courses, the characteristics of calcium signals obtained from in vitro and in vivo activity recordings in this study, such as transient accumulation of intracellular Ca^2+^ with peaks of activation of ~20 s upon magnetic stimulation, are of potential interest for future studies investigating magnetogenetics mechanisms. Bidirectional control of neuronal activity through the unimolecular fusion proteins described here that control the relationship between the TRPV1 mutants and the ferritin nanobody should also provide advantageous tools to further analyze these possible mechanisms.

In summary, the present study demonstrates that magnetogenetic gene therapy efficiently regulates basal ganglia circuitry. A single AAV encoding the activating magnetogenetic construct restricted to iSPNs elicited a freezing phenotype in transgenic mice. Circuit-specific magnetogenetic activation of iSPNs was replicated in WT mice using AAVretro to deliver Cre to a specific neuronal population, while expression of a novel inhibitory magnetogenetic construct within the STN reduced activity with consequent motor improvement in hemiparkinsonian mice. Our results provide a bidirectional toolkit for magnetogenetic regulation to study specific central or peripheral neural circuits, while further development of the technology may facilitate translation into amenable human conditions where noninvasive and immediate modulation of neuronal activity could have therapeutic benefit.

## MATERIALS AND METHODS

### Ferritin nanobody generation

#### Immunization of a llama

A llama was injected subcutaneously with human spleen ferritin (250 μg) (Lee Biosolutions, MO) on days 0, 7, 14, 21, 28, and 35. Gerbu LQ#3000 was used as an adjuvant. On day 40, anticoagulated blood was collected for lymphocyte preparation.

#### Construction of a VHH library

A variable heavy domain of heavy chain (VHH) library was constructed and screened for the presence of antigen-specific nanobodies. To this end, total RNA from peripheral blood lymphocytes was used as a template for first-strand cDNA synthesis with an oligo(dT) primer. Using this cDNA, the VHH encoding sequences were amplified by polymerase chain reaction (PCR), digested with Pst I and Not I, and cloned into the Pst I and Not I sites of the phagemid vector pMECS. A VHH library of about 108 independent transformants was obtained. About 87% of transformants harbored the vector with the right insert size.

#### Isolation of antigen-specific nanobodies

The library was subject to three consecutive rounds of panning on solid-phase coated human ferritin (200 μg/ml, 20 μg per well). The enrichment for human ferritin-specific phages was assessed after each round of panning by comparing the number of phagemid particles eluted from antigen coated wells with the number of phagemid particles eluted from negative control wells blocked without antigen. These experiments suggested that the phage population was enriched about 20-fold, 20-fold, and 8 × 10^2^-fold for human ferritin-specific phages after first, second, and third rounds of panning, respectively. A total of 190 colonies from first and second rounds were randomly selected and analyzed by ELISA for the presence of human ferritin-specific nanobodies. This first round of ELISAs was used to screen nanobodies in crude periplasmic extracts, and the relative intensity of the ELISA signal may not reflect the relative quality of the nanobodies. In these experiments, the differences in ELISA signals between different nanobodies may be related to variables such as the amount of nanobody, rather than to nanobody quality such as affinity or actual yield. Of these 190 colonies, 133 colonies scored positive for the presence of nanobodies to human ferritin in this assay (46 and 87 from first & second rounds, respectively). The human ferritin used for panning and ELISA screening was the same as the one used for immunization. On the basis of sequence data, the ELISA-positive colonies represented 59 different nanobodies belong to 22 different CDR3 groups.

Next, the library was subject to three consecutive rounds of panning on solid phase coated with mouse ferritin purified from the mouse liver (200 μg/ml, 20 μg per well). The enrichment for mouse ferritin-specific phages was assessed as above. These experiments suggested that the phage population was enriched about 10-fold, 10-fold, and 5 × 10^2^-fold for mouse ferritin-specific phages after first, second, and third rounds of panning, respectively. A total of 190 colonies from second & third rounds were randomly selected and analyzed by ELISA for the presence of mouse ferritin-specific nanobodies in their periplasmic extracts (ELISA using crude periplasmic extracts including soluble nanobodies). Of these 190 colonies, 54 colonies scored positive in this assay (1 and 53 from second & third rounds, respectively). On the basis of sequence data, the ELISA-positive colonies represented 19 different nanobodies belonging to six different CDR3 groups. Two nanobody CDR3 groups bound both human and mouse ferritin.

### Purification of recombinant nanobody protein

pMECS vectors encoding anti-ferritin nanobodies were transformed into electrocompetent *Escherichia coli* WK6 cells. Colonies were picked and grown in 10 to 20 ml of LB + ampicillin (100 μg/ml) + glucose (1%) at 37°C overnight. One milliliter of preculture was added to 330 ml of TB-medium (2.3 g KH_2_PO_4_, 16.4 g K_2_HPO_4_·3H_2_O, 12 g of tryptone, 24 g of yeast, and 4 ml of 100% glycerol) supplemented with ampicillin (100 μg/ml), 2 mM MgCl_2_, and 0.1% glucose and grown at 37°C. Nanobody expression was induced by addition of isopropyl-β-d-thiogalactopyranoside (1 mM). The culture was incubated at 28°C with shaking overnight. The cells were pelleted for 8 min at 8000 rpm and resuspended in 12 ml of TES [0.2 M Tris (pH 8.0), 0.5 mM EDTA, and 0.5 M sucrose] before shaking for 1 hour on ice. A further 18 ml of TES (diluted fourfold in water) was added and incubated for a further hour on ice with shaking then centrifuged for 30 min at 8000 rpm at 4°C. The supernatant containing the nanobody proteins was then purified by immobilized metal affinity chromatography using HIS-Select Cobalt Affinity Gel (Sigma-Aldrich, H8162) according to the manufacturer’s instructions. The amount of protein was estimated at this point by OD280 (optical density at 280 nm) measurement of the eluted sample. Fourteen nanobodies were successfully purified.

### Characterization of nanobody affinity to human ferritin by ELISA

ELISA plates were coated with human spleen ferritin (1 μg/ml; Lee Biosolutions, MO) in phosphate-buffered saline (PBS) overnight at 4°C. Plates were then washed five times with washing buffer (PBS + 0.05% Tween 20), followed by blocking with PBS + 1% bovine serum albumin (BSA) at room temperature (RT) for 2 hours. Serial dilutions of nanobodies or BSA (100 μl) were added to the ELISA plates and incubated for 2 hours at RT then washed five times with buffer. Anti-HA-HRP (horseradish peroxidase) antibody (100 μl; Miltenyi Biotec, 130-091-972; 1:1000 dilution) was added to each well and incubated for 1 hour at RT before a further five washes. The 3,3ʹ,5,5ʹ tetramethylbenzidine (TMB) substrate (100 μl) was added to each well, and the reaction stopped by addition of 100 μl of 2 M H_2_SO_4_ after 10 min. OD450 was measured using a microplate reader (BMG Labtech CLARIOstar plate reader).

### Characterization of anti-ferritin nanobodies by immunoprecipitation

Nanobody sequences were amplified by PCR with the specific primers (NbFt-F: gcaagatctgccaccatggcc CAGGTGCAGCTGCAGGAG; NbFt-R: gcaaagcttggatccAGCGTAATCTGGAACATCGTATGGGTA tgcggccgctgagga) and subcloned into the retroviral vector pMSCV (Clontech, Takara Bio, United States). HEK-293T cells were grown in 10-cm plates and cotransfected with plasmids expressing GFP-mFerritin and anti-ferritin nanobody clones using Polyethylenimine (PEI) (DNA:PEI = 1:3). Cells were harvested after 48 hours and lysed with 500 μl of lysis buffer [50 mM Tris, (pH 7.5), 300 mM NaCl, 1 mM EGTA, 1 mM EDTA, 1% NP-40, and 0.1% SDS] and protease inhibitor (complete EDTA-free, Roche) then incubated for 15 min with agitation at 4°C before centrifugation at 14,000 rpm for 20 min. The supernatant was transferred to a new tube with 30 μl of HA.11 antibody conjugated agarose beads (BioLegend, 900801) added to each sample. The beads and supernatant were incubated at 4°C overnight with rotation and then spun at 3000 rpm for 2 min. Beads were then washed three times with lysis buffer before proteins were eluted with 20 μl of 4x Laemmli buffer and heated at 95°C for 5 min. Eluted proteins were examined by SDS–polyacrylamide gel electrophoresis (PAGE). Samples were loaded onto a 10-well SDS-PAGE gel and run for 10 to 15 mm then fixed in 46% methanol/7% glacial acetic acid for 1 hour. The gel was then stained for 1 hour in 0.1% Coomassie blue R-250 in 46% methanol/7% glacial acetic acid before destaining in ultrapure water. Bands were then excised and analyzed by mass spectrometry.

### Mass spectrometry

Proteins were reduced (10 mM dithiothreitol, EMD Millipore) and alkylated (30 mM iodoacetamide, Sigma-Aldrich), followed by digestion with Endoproteinase LysC (Wako Chemicals) and trypsin (Promega). Reaction was halted by addition of trifluoracetic acid, and peptides were solid phase extracted (*46*) and analyzed by reversed-phase nano-LC-MS/MS (Dionex 3000 coupled to a Q-Excative Plus, Thermo Fisher Scientific). Data were quantified and searched against a UniProt human database concatenated with a mouse GFP-Ferritin sequence using ProteomeDiscoverer 1.4/Mascot 2.5. Oxidation of methionine and protein N-terminal acetylation were allowed as variable modifications. Cysteines were considered as fully carbamidomethylated. Two missed cleavages were allowed. Peptide matches were filtered using a percolator (*47*) calculated false discovery rate of 1%. The average area of the three most abundant peptides per protein were used as a proxy for protein abundance (*48*). For the highly homologous ferritin heavy proteins, only peptides unique to either the mouse or the human forms were used in access abundance. Peptides specific to the N-terminal GFP moiety of the overexpressed mouse ferritin were not used to calculate the abundance of mouse ferritin but were recorded separately.

### Isothermal titration calorimetry

We performed an isothermal titration calorimetry assay using a MicroCal Auto-iTC200 instrument (Malvern Panalytical). In each experiment, 10 μM human spleen ferritin in the cell was titrated with 75 μM NbFt2 in the syringe at 25°C. The titration sequences included a single 0.4-μl injection, followed by 19 injections of 2 μl each, with a 150-s interval between injections, stirring rate of 750 rpm, and reference power of 10 μcal/s. Data were analyzed with the Origin analysis software.

### Generation of constructs

The expression vectors for calcium-dependent release of insulin and SEAP, pCMV-TRPV1^Ca2+^ were generated as previously described (*20*, *21*). To generate the Ca^2+^-dependent SEAP reporter, the SEAP coding fragments were obtained by cutting pYSEAP (Addgene, catalog no. 37326) with Hind III and Hpa I and inserted into pSRE-CRE-NFATinsulin (*21*) at the Hind III and Hpa I sites. To express Nb-FT in mammalian cells, a PGK promoter-driven mCherry expression cassette and the WPRE element were inserted into pMSCV (Clontech, Takara Bio) at the Xho I and Cla I sites to generate an empty vector (pMSCV-mCherry-WPRE). The anti-ferritin nanobody coding sequences were amplified from phage display plasmids with specific primers: 5′gcaagatctgccaccatggccCAGGTGCAGCTGCAGGAG and 5′gcaaagctt ggatccAGCGTAATCTGGAACATCGTATGGGTAtgcggccgctgagga, digested with Bg lII and Hind III and ligated into pJFXY21 at the Bg lII and Hind III sites. The resulting plasmids were named pMSCV-Nb-Ft-2, -9, - 10, -14, and -17-TRPV1^Ca2+^. The Nb-GFP fragment was amplified from MSCV-αGFP-TRPV1-2AGFPferritin (*16*) with specific primers: 5′gcaagatctgccaccatggcc CAGGTGCAGCTGCAGGAG and 5′ tcagacgtcggccactgcggccgcTGAGGAGACGGTGACCTGGGTC, digested and subcloned into pNb-Ft-2-TRPV1^Ca2+^ at the Bg lII and Not I sites to generate plasmid pNb-GFP-TRPV1^Ca2+^. The pMSCV-Nb-GFP-TRPV1^Ca2+^-T2A-GFP-mFerritin plasmid was generated by subcloning the Nb-GFPTRPV1^Ca2+^-T2A-GFP-mFerritin fragment cut from MSCV-αGFP-TRPV1-2A-GFPferritin (*16*) with Not I and Eco RI into a modified pMSCV-WPRE vector at the Not I and Mfe I. NbFt sequences were amplified from plasmids pMSCV-Nb-Ft-2, -9, -10, -14, and -17-TRPV1^Ca2+^ with specific primers: 5′ gtgatgcatGCCACCATGGCCCAGGTGCAG and 5′ gatgctagcgccAGCGTAATCTGGAACATCG, digested with Nsi I and Nhe l and inserted into pJFXY16 at the Nsi I and Avr II sites. The resulting constructs were named pMSCV-Nb-Ft-2, -9, -10, -14, and -17-TRPV1^Ca2+^-T2A-GFP-mFerritin. The pMSCV-GFP-mFerritin vector was created by digesting pMSCV-Nb-GFP-TRPV1^Ca2+^-T2A-GFP-mFerritin with Bg lII to remove the NbGFP-TRPV1-T2A fragment and self-ligated.

To make pAAV-hSyn-Nb-FT-2-TRPV1^Ca2+^, the 2981-bp PCR product containing Nb-FT-2-TRPV1^Ca2+^ was amplified from pMSCV-Nb-Ft-2-TRPV1^Ca2+^-T2A-GFP-mFerritin using primers 5’AGCGCAGTCGAGAAGGTACCGGATCCCCCGGTCGCCACCACTAGTATGGCCCAGGTGCAGCTGC and 5’-TTATCGATAA-GCTTGATATCGAATTCTTACTTCTCCCCTGGGACCATG, digesting with Bam HI and Eco RI and cloning into Bam HI/Eco RI-digested pAAV-hSyn-mCherry (*50*).

To make pAAV-hSyn-RCaMP, a 1441-bp PCR product containing jRGECO1a, a red fluorescent calcium sensor protein (RCaMP) was amplified from pAAV.Syn.Flex.NES-jRGECO1a.WPRE.SV40 (Addgene, catalog no. 100853) using primers 5’-ACTAGTAT-GCTGCAGAACGAGCTTGC and ACCGGTCTACTAGTCTCAATTGTCACTTCGCTGTCATCATTTGT, digested with Spe I, and cloned into pAAV-hSyn-DIO-MCS in the forward direction so that RCaMP could be expressed in the absence of Cre recombinase. pAAV-hSyn-DIO-MCS is pAAV-hSyn-DIO-hM3D(Gq)-mCherry (Addgene, catalog no. 44361), which previously had the Bsr GI/Nhe I region containing hM3D(Gq)-mCherry replaced with the following multiple cloning site: TGTACAACTAGTACCGGTTCGCGAGCATGCCCTAGGGCTAGC.

### Cell culture and in vitro studies

HEK-293T [ATCC CRL-3216, mycoplasma testing and short tandem repeat (STR) profiling performed by American Type Culture Collection (ATCC)] were cultured in Dulbecco’s modified Eagle’s medium (DMEM) with 10% fetal bovine serum (FBS) (Gibco, Carlsbad, CA) at 37°C and 5% CO_2_. Neuro 2A cells (ATCC CCL-131, mycoplasma testing and STR profiling performed by ATCC) were grown in Eagles’ minimum essential medium with 10% FBS (Gibco) at 37°C and 5% CO_2_.

#### Immunocytochemistry studies

HEK-293T or Neuro 2A cells were cultured on 12-mm cover glass (Fisher Scientific, Pittsburgh, PA) coated with fibronectin (Sigma-Aldrich, catalog no. F1141). Cells were transfected with constructs expressing pAAV-hSyn-Nb-FT-2-TRPV1^Ca2+^ or pCMV-TRPV1^Ca2+^ 24 hours after plating using X-tremeGENE 9 DNA Transfection Reagent (MilliporeSigma) according to the manufacturer’s specifications. Cells were stained 48 to 72 hours after transfection.

#### Magnet treatment studies using calcium-dependent reporters

HEK-293T cells were cultured on 12-mm cover glass and transfected with pMSCV-Nb-Ft-TRPV1^Ca2+^ or pMSCV-Nb-GFP-TRPV1 and GFP-mFerritin and either calcium-dependent SEAP construct (*53*) or calcium-dependent insulin construct (*8*). Holotransferrin (2 mg/ml, Sigma-Aldrich) was added to cells 24 hours after transfection. Twenty-four hours prior to the study, cells were placed in 1% FBS medium at 32°C to ensure minimal activation of TRPV1 and calcium-dependent pathways. Cells were incubated in 300 μl of calcium imaging buffer at RT (control) or in an oscillating magnetic field (465 kHz, 30 mT) at RT. After 60 min, the supernatant was removed, spun to remove cells, and assayed for SEAP or insulin. Screening studies with SEAP production were repeated twice with four replicates. Validation studies with calcium-dependent insulin production were repeated at least three times with at least three replicates.

#### Calcium imaging studies

Neuro2A cells were cultured on cover glass and transfected with pAAV-hSyn-Nb-FT-2-TRPV1^Ca2+^ with or without pMSCV-GFP-mFerritin as described above with holotransferrin added 24 hours after transfection. Cells were placed at 32°C 24 hours before testing. Cells were loaded with 3 μM Fluo-4 (Invitrogen) in the presence of 500 μM sulfinpyrazone (Sigma-Aldrich) for 45 to 60 min at 32°C then washed and incubated for 15 to 30 min in sulfinpyrazone in PBS. HEK-293T cells were cultured on cover glass and transfected with pAAV-hSyn-RCaMP alone or combined with pAAV-hSyn-Nb-FT-2-TRPV1^Ca2+^ or pCMV-TRPV1^Ca2+^. Cells were then placed in glass-bottom dishes with calcium imaging buffer at 29° to 31°C for imaging. Imaging was performed using a Deltavision personal DV imaging system (Applied Precision Inc., Issawaq, WA) equipped with a custom-made ceramic lens and softWoRx imaging station. All other calcium imaging experiments were performed on an inverted Zeiss Axio Observer Z1 microscope (Carl Zeiss, Oberkochen GER). Cells were imaged before and during RF treatment, before and during application of a permanent magnet, before and after treatment with capsaicin (1 μM; Sigma-Aldrich). Imaging was performed on at least three occasions for each condition. Image analysis was performed using ImageJ. Briefly, regions of interests (ROIs) were selected for background or cells and the mean intensity was calculated for every ROI in each image. Calcium responses were calculated as the change in Fluo-4 fluorescence normalized to the baseline fluorescence (Δ*F*/*F*_0_) for each cell, and these data were plotted as the mean across all cells at each time point. In addition, we calculated the peak Δ*F*/*F*_0_ value for each cell, irrespective of when this peak occurred.

### Immunocytochemistry of cultured cells

Immunocytochemistry (ICC) and immunohistochemistry (IHC) were used to confirm cell surface expression of TRPV1. Live cells were incubated with rabbit anti-TRPV1 (extracellular) polyclonal antibody (Thermo Fisher Scientific, catalog no. PA5-77361) (1:50) in culture medium for 10 min at 37°C followed by five washes with RT culture medium. Cells were then incubated with goat anti-rabbit Alexa Fluor 568 or 633 (1:1000) for 10 min at 32°C followed by a further five washes with RT culture medium. Cells were then fixed in 3.7% paraformaldehyde (PFA) in Hanks’ balanced salt solution (HBSS) for 30 min at RT and washed five times in HBSS before mounting using Fluoromount with 4′,6-diamidino-2-phenylindole (DAPI) (Southern Biotech, Birmingham, AL). Images were acquired using a Zeiss LSM 880 inverted confocal microscope (Carl Zeiss, Oberkochen GER).

### Electrophysiology recordings of N2A cells expressing the pAAV-hSyn-Nb-FT-2-TRPV1^Ca2+^ construct

Neuro2A cells were cultured on uncoated petri dishes and were transfected with pAAV-hSyn-Nb-FT-2-TRPV1^Ca2+^ (as above). Electrophysiological recordings were performed 24 to 48 hours after transfection in a bath solution consisting of 10 mM Hepes (pH 7.4), 140 mM NaCl, and 5 mM EGTA and were imaged using a Nikon eclipse DIC microscope at ×20 magnification. Pipettes of borosilicate glass (Sutter Instruments, BF150-86-10) were pulled to ~3 to 5 molecular weight resistance with a micropipette puller (Sutter Instruments, P-97) and polished with a microforge (Narishige, MF-83). The pipette was filled with an identical bath solution. Recordings were obtained with an Axopatch 200B amplifier (Molecular Devices), filtered at 1 kHz, and digitized at 10 kHz (Digidata 1440, Molecular Devices). Recordings were made in the outside-out excised patch configuration after gigaseals were obtained and currents were recorded by voltage ramp protocols from −100 to +100 mV with and without 10 mM capsaicin addition by perfusion (ALA Scientific VM8 manifold perfusion system).

### Generation of AAV constructs

pAAV-JET-DIO-MCS/pLP362 is the backbone for the AAV vectors used to make pAAV-JET-DIO-mCherry, pAAV-JET-DIO-Nb-FT-TRPV1^Ca2+^, pAAV-JET-DIO-Nb-Ft-TRPV1^Cl−^, and pAAV-JET- DIO-TRPV1^Ca2+^ and was made by replacing the Bsr GI/Nhe I region of pAAV-hSyn-DIO-hM3D(Gq)-mCherry (Addgene, catalog no. 44361) with a multiple cloning site (TGTACAACTAGTACCGGTTCGCGAGCATGCCCTAGGGCTAGC) and inserting a 242-bp PCR product containing the JET promoter (*55*) amplified using primers 5′- CTGCGGCCGCACGCGTGTACCATTGACGAATTCGGGCG and 5′- ACTCTAGAGGATCCGGTACCTGTCAAGTGACGATCACAGGG into the Mlu I/Kpn I sites.

To make pAAV-JET-DIO-mCherry/pLP388, the mCherry open reading frame was amplified using primers 5′-ATTACCGGTGTTAACATGGTGAGCAAGGGCGAGGA and 5′-TAAACCGGTCTTAAGTTACTTGTACAGCTCGTCCATG and cloned into AgeI-digested pAAV-JET-DIO-MCS with the mCherry open reading frame in the reverse orientation to the JET promoter.

pAAV-JET-DIO-Nb-FT-TRPV1^Ca2+^/pLP363 was made by ligating a 2943-bp Spe I/Eco RV fragment containing NbFT-TRPV1^Ca2+^ (*33*) to Nru I/Avr II digested pAAV-JET-DIO-MCS.

The 865-bp Hind III/Tth111 I fragment of pAAV-JET-DIO-Nb-FT-TRPV1^Ca2+^ was replaced with a 445-bp Hind III/Tth111 I fragment of synthesized DNA (Azenta Life Sciences, NJ) lacking the Nb-Ft sequence to make pAAV-JET-DIO-TRPV1^Ca2+^/pLP452.

To make pAAV-JET-DIO-Nb-Ft-TRPV1^Cl−^/pLP375, primers 5′-TCCATGGTGTTCTCCCTGGCAATGGGCTGGACCAAC-ATGCTCT and 5′-AGACTAGTGTTATTTCTCCCCTGGGACCA were used to remove one Nco I site and amplify an 876-bp Nco I fragment of TRPV1^mutant^ encoding the I679K mutation (*10*), which was cloned into Nco I-digested pAAV-JET-DIO-NbFT-TRPV1^Ca2+^.

pAAV-CMV-Cre-GFP was obtained from Addgene (catalog no. 68544).

### Cell culture and AAV preparation

HEK-293 cells (ATCC CRL-1573) were cultured in DMEM (Gibco), supplemented with 10% (v/v) FBS (Sigma-Aldrich) and 1% (v/v) penicillin-streptomycin (Gibco), at 37°C in 95% humidified air and 5% CO_2_. Vector stocks were prepared by packaging the plasmids into rgAAV2 or mixed serotype AAV1/2 particles using a calcium phosphate transfection system as described previously (*56*). Cells were harvested and lysed at 72 hours after transfection. The vectors were purified using iodixanol gradient and dialyzed against PBS with 2 mM MgCl_2_. AAV titers were determined by quantitative PCR (qPCR) using Syber Green chemistry and with primers to the WPRE fragment of the AAV backbone.

### Quantitative real-time PCR

To titer our purified viral vectors, AAV were processed as previously described (*56*) and viral genomes quantified via qPCR using Fast SYBR green master mix (Applied Biosystems, catalog no. 4385612) on the AB 7500 FAST Real Time PCR platform (Applied Biosystems) and primers to the WPRE element: WPRE-Fw: 5′-GGCTGTTGGGCACTGACAAT-3′; WPRE-Rev: 5′-CTTCTGCTACGTCCCTTCGG-3′. The relative number of full viral particles was calculated using the standard curve method by normalizing to known standard samples.

### Animals

Male mice were housed two to five per cage and kept at 22°C on a reverse 11 a.m. (light)/11 p.m. (dark) cycle, with standard mouse chow and water provided ad libitum throughout the duration of the study. All animal procedures were approved by the Institutional Animal Care and Use Committee (IACUC) of Weill Cornell Medicine and were in accordance with the National Institutes of Health (NIH) guidelines (IACUC no. 2009-0026).

The following mice were used in these studies: (i) WT C57BL/6J mice, obtained from the Jackson Laboratory (Jax.org; strain no. 000664); (ii) 129S-Pitx2tm4(cre)Jfm/Mmucd (PitX2-Cre; MMRRC stock no. 000126-UCD); and *B6.FVB(Cg)-Tg (Adora2a-cre) KG139Gsat/Mmucd* (A2a-Cre; MMRRC stock no. 036158-UCD).

### Stereotactic surgery

All stereotactic surgical procedures were performed on 8- to 12-week-old mice, weighing 20 to 25 g, under a mixture of ketamine/xylazine anesthesia. Ketamine (Butler Animal Health Supply) and xylazine (Lloyd Laboratories) were administered intraperitoneally at concentrations of 110 and 10 mg/kg body weight, respectively. After the induction of anesthesia, the animals were placed into a stereotactic frame (David Kopf Instruments). All infusions were performed using a 10-μl stereotactic syringe attached to a microinfusion pump (World Precision Instruments) at a rate of 0.1 to 0.4 μl/min. To prevent reflux, after each infusion, the injection needle was left in place for 5 min, withdrawn a short distance (0.3 to 0.5 mm), and then left in the new position for an additional 2 min before removal with a 33-gauge needle.

To generate 6-OHDA lesioned mice, animals were injected with a total volume of 0.6 μl of 6-OHDA hydrobromide (Sigma-Aldrich) in PBS with 0.1% ascorbate unilaterally into the medial forebrain bundle (MFB) at a concentration of 2.5 mg/ml and an infusion rate of 0.1 μl/min with a 10-μl Hamilton syringe with a 30-gauge needle. The coordinates for the injection were AP −1.1 mm, ML −1.1 mm, and DV −5.0 mm relative to bregma and the dural surface. Before lesion surgery, the norepinephrine reuptake inhibitor desipramine (25 mg/kg, ip) was administered via intraperitoneal injection at least 30 min before 6-OHDA infusion to protect neostriatal and cerebellar noradrenergic neurons from the toxin-induced damage. Mice were allowed 4 to 6 weeks of recovery before being subjected to the apomorphine-induced a behavioral test to estimate the extent of dopamine depletion in the substantia nigra (see following section).

For experiments in PitX2-Cre mice, AAV1/2 vectors were injected into the STN unilateral to the lesioned side (AP −2.0 mm, ML −1.7 mm, and DV −4.7 mm from bregma) of 6-OHDA mice. For experiments in A2a-cre mice, AAV1/2 vectors were injected bilaterally into the dorsal striatum (AP +0.5 mm, ML ±2.3 mm, and DV −3.5 mm from bregma). For experiments in WT mice, AAV1/2 vectors were injected bilaterally into the dorsal striatum (AP +0.5 mm, ML ±2.3 mm, and DV −3.5 mm from bregma) and AAV2retro into the Gp (AP +0.5 mm, ML ±2.3 mm, and DV −3.5 mm from bregma). All craniotomies to inject AAV vectors were performed at a rate of 0.4 μl/min using a 10-μl WPI syringe with a 33-gauge needle. A total of 2 × 10^11^ genomic particles/μl (2 μl in PBS) of vectors were injected.

The mice were allowed a 6- to 8-week recovery before being subjected to the behavioral tests during which maximal transgene expression from AAV vectors is achieved. At the conclusion of the experiments, injection site accuracy within the targeted brain region was determined by IHC for the specific transgene expressed by the AAV vectors, and mice with mistargeted injections were excluded from analysis before their data were unblinded.

### Sources of magnetic field

#### Direct magnetic field

The magnetic field for in vivo studies was generated by the superconducting electromagnetic MRI field from a Siemens 3.0 Tesla PRISMA MRI Scanner (Siemens Healthcare). The magnetic field strength was measured and mapped using a gaussmeter (F.W. BELL MODEL 5080). High DMF was defined as a region with a strength of 0.5 to 1.3 T, and low DMF as 0.1 to 0.27 T.

#### Transcranial magnetic stimulation

A butterfly C-B60 coil (165 x 85 x 19 mm/inner diameter 35 mm/outer diameter 75 mm) was used to deliver magnetic field during in vivo studies. Biphasic stimulation pulses were delivered by a waveform generator (MagPro100x with MagOption) with the following settings: 20% machine capacity (~600 mT), twin/dual mode 50-Hz stimulation, 500-μs rise time, 1-ms interpulse interval, 10 pulses in train, number of trains 20, and intertrain interval 1 s for 20 s. This set up was determined based on trials where small percent increments in machine capacity were performed until a freezing phenotype was observed or until an electric shock–induced response was recorded. In those trials, 25% of TMS capacity caused electric shock–induced responses such as twitching (movies S3 to S5). Field mapping is available upon request to MagVenture (https://magventure.com).

### Behavioral assessments

All behavioral tests were run during the dark phase of the mouse daily cycle. The experiments were performed by an examiner blinded to treatment groups.

#### Apomorphine induced rotations

To test for 6-OHDA lesion efficiency in PitX2-Cre mice, rotational behaviors were performed. In brief, mice were placed in a body harness connected to a transducer/swivel in a bowl-shaped testing arena (rotometer). Full 360° clockwise and counterclockwise rotations were measured over a 45-min period after drug injection for apomorphine-induced rotations. Apomorphine (Sigma-Aldrich) was dissolved in saline solution, 0.9% NaCl, and 0.1% ascorbate and administered at a dose of 0.25 mg/kg via subcutaneous injection. Mice we randomized based on the net number of contralateral rotations before receiving STN injections of the inhibitory magnetogenetic construct.

To test the effect of our AAV.Nb-Ft-TRPV1^Cl−^ vectors on inhibiting STN neuronal activity, rotational behaviors were performed inside a 3T MRI room in a sound-insulated, ventilated Styrofoam chamber that contained a plastic arena of 26.5 x 26.5 x 26.5 cm. Video recordings of apomorphine induced rotations were obtained beginning 10 min after 0.25 mg/kg via subcutaneous injections for 5 min outside the magnetic field followed by 5 min inside the magnetic field (500 mT to 1.3 T) and another 5 min outside the magnetic field in the start position using a magnetic field compatible camera (MRC HiSpeed camera, https://mrc-systems.de/en/products/mr-compatible-cameras#12m-camera). Upon the conclusion of behavioral studies, nigral lesion efficiency by 6-OHDA was determined by IHC for the tyrosine hydroxylase (TH) protein as a marker for dopaminergic neurons in the substantia nigra. Mice with mistargeted injections were excluded from analysis before their data were unblinded.

#### Open-field arena

Locomotor behavior in an open-field arena was performed inside a 3T MRI room using the same chambers and video recording system as described above for apomorphine-induced rotations. For 2 days prior to testing, each mouse was habituated in the open-field box for 10 min to reduce any possible confounding effect of a new environment. On the day of testing, each mouse was placed in the center of the open field, and spontaneous horizontal and vertical behaviors were recorded during 5-min sessions of pre-DMF, during DMF, and post-DMF. To limit effects of cage movement on animal anxiety and locomotion, the MRI table with the cage was slowly moved into and out of position over roughly 1 min for the DMF and post-DMF conditions, respectively.

#### Noldus EthoVision

EthoVision XT14 was used to analyze offline rotational behaviors and open-field videos. The automatic animal detection with deep learning settings was used to track center-to-nose and center-to-tail points in defined arenas. Video recordings were used to analyze the following locomotion and rotational parameters: (i) distance traveled, (ii) activity percentage (%), and (iii) rotation >90° clockwise and counterclockwise. Tracking data and heatmaps were graphed with EthoVision analysis tools.

#### TRACKER

TRACKER video analysis and modeling tool (TRACKER 6 free software, https://physlets.org/tracker/) was used to track precise motor responses, including freezing and ambulation time. Each second of the recordings was divided into five frames to track the nose, center, fore and hind paws, and tail every two frames resulting in 150 positions measured per minute. Spatial motion in the *Y* axis, referred as “change of location,” was also analyzed as a function of time. Freezing of gait was defined as a continuous period of 1-s (or >5 frames) period with no change of spatial position of the head, center, or extremities.

### IHC of brain sections

On completion of all assessments, mice were deeply anesthetized with sodium pentobarbital (150 mg/kg, ip) and transcardially perfused with 4% PFA. Brains were extracted and post-fixed overnight in 4% PFA, cryoprotected in 30% sucrose, and cut into 30-μm sections using a vibratome (Leica Microsystems). Free-floating sections were treated with various antibodies to visualize proteins of interest using immunofluorescence labeling, including TH to quantify the extent of nigral neurodegeneration, and mCherry or HA to estimate the transduction efficiency from our AAV vectors. The following primary antibodies were used: rabbit anti-RFP to detect mCherry (Rockland, catalog no. 600-401-379; dilution 1:1000), sheep anti-TH (Millipore, catalog no. AB1542; dilution 1:1000), rabbit anti-HA (Sigma-Aldrich, catalog no. H6908, dilution1:500), and rat anti–c-fos (Synaptic Systems, catalog no. 226-017, dilution 1:1000). Alexa Fluor–conjugated fluorescent secondary antibodies (Molecular Probes) were obtained from Life Technologies and used at a 1:1000 dilution. For *c-fos* analyses, mice were exposed to the DMF (MRI magnet, 500 mT to 1.3 T) for 20 min prior to being transcardially perfused as described above. All mice that were included in the *c-fos* data were perfused within 1 hour following DMF exposure.

### RNAScope in situ hybridization

Thirty micrometer free-floating brain sections were mounted in PBS onto charged Superfrost slides (Fisher Scientific) and were allowed to air dry for ~2 hours. The experiments were performed as per the manufacturer’s instructions (ACDbio). The incubations at 40° and 60°C took place in the HybEZ oven using the EZ-Batch system (ACDbio). Sections were first baked at 60°C for 1 hour and post-fixed in 4% PFA for 15 min at 4°C. After the dehydration steps, slides were dried at 60°C for 15 min, treated with hydrogen peroxidase (catalog no. 322335, ACDbio) at RT for 10 min, and dried again at 60°C for 15 min before performing the retrieval step. Sections were incubated in target retrieval buffer (catalog no. 322000, ACDbio) for 10 min at 100°C and washed two times with double deionized water (ddH_2_O). After 3-min incubation in 100% ethanol and drying at 60°C for 10 min, sections were then incubated in Protease III (catalog no. 322337, ACDbio) at 40°C for 30 min, washed five times with ddH_2_O, and incubated for 2 hours at 40°C with probes including Mm-Drd2-C1, Mm-PitX2-C1, Mm-Drd1-C3, and mCherry-C4. A slide for either positive or negative control probes were also included (catalog no. 321811 and 321831, respectively, ACDbio). Two washes occurred in between the following 40°C incubations with wash buffer (catalog no. 310091). Sections were incubated for 30 min with AMP1, 30 min with AMP2, and 15 min with AMP3 (kit catalog no. 323110). Next, sections were incubated in HRP C1 for 15 min and Opal 520 (1:1500; catalog no. OP-001001) in TSA buffer (catalog no. 322809). Sections were next incubated in an HRP blocker for 15 min. Following sections were incubated with HRP C2 in combination with Opal 650 (1:1500; catalog no. OP-001003) and HRP C4 in combination with Opal 570 (1:1500; catalog no. OP-001005). Slides were cover slipped with Prolong Gold containing DAPI (ACDbio).

### PET/CT animal imaging studies

All animal imaging experiments were performed in the Citigroup Biomedical Imaging Center (CBIC, Weill Cornell Medicine). For baseline experiments, mice were fasted for 12 hours prior to imaging followed by intraperitoneal injection of ~500 μCi ^18^F-FDG, and residual dose was measured. One hour after injection, mice were anesthetized with isoflurane and scanned for 1 hour in a micro-PET/CT scanner (Siemens Inveon). For DMF experiments, mice were exposed to a 3T MRI (DMF = 500 mT to 1.3 T) for a total of 25 min after ^18^F-FDG intraperitoneal administration and scanned following the same protocol as for baseline experiments. Following imaging, scans were histogrammed and reconstructed using the Siemens Inveon Acquisition Workplace (Siemens Medical Solutions, Knoxville, TN, United States) and analyzed in DICOM format. MRICron and MATLAB were used to do a PET-CT coregistration, and the PMOD Fusion tool (version 3.6, PMOD Technologies Ltd., Zurich, Switzerland) was used for rigid transformations [manually coregistered via rigid transformations (translations and rotations)], matching with MRI mouse template, normalization with Mirrione mouse brain atlas, and ROI analysis. All analyses were conducted in a blinded manner and were adjusted for mouse weight, ^18^F-FDG final dose (^18^F-FDG_initial dose_ − ^18^F-FDG_residual dose_), as well as relative to regions that do not express D2-type receptors such as the cerebellum. The right and left dorsal striata were averaged for each animal and MBq/ml or percent injected dose per gram (%ID/g) were calculated to plot data. Acquired PET-CT imaging data were correlated with locomotor data obtained on a previous DMF exposure session.

### In vivo fiber photometry

Fiber photometry was performed to measure in vivo calcium-dependent activity dynamics during DMF/TMS stimulation. Cre-dependent AAVs expressing GCaMP6s (AAV9-Syn-DIO-GCaMP6s, Addgene, catalog no. 100845-AAV9) and Nb-Ft-TRPV1^Ca2+^-HA/mCherry were injected bilaterally into the dorsal striatum of A2a-Cre transgenic mice to restrict expression of GCaMP6s and NbFt- TRPV1^Ca2+^-HA or mCherry to D2 iSPNs. Two weeks following AAV injection, a 400-μm-diameter optical fiber (Doric, MFC_400/430-0.48_5.5mm_ZF2.5(G)_FLT, catalog no. B280-4418-5) was implanted in the dorsal striatum 0.5 mm above the AAV injection site (AP +2.00 mm, ML −0.25 mm, and DV −2.15 mm) and was secured with Metabond. Before behavioral testing, mice were habituated for 2 days to a patch cord attached to the implanted optical fiber for 3-min sessions.

During behavioral testing, fiber photometry recordings were taken for 2 min before, during, and post-magnetic stimulation. Behaviors were performed and recorded using the same settings as described above for open-field arena locomotor quantification. Following behavioral testing, fluorescence microscopy was used to confirm GCaMP6s expression and optical fiber placement. To excite GCaMP6s, light from a 470-nm light-emitting diode (Thorlabs, M470F3) modulated at a frequency of 521 Hz was passed through a filter (Semrock, FF02-472/30), reflected by a dichroic (Semrock, FF495-Di03), and coupled to a 0.48–numerical aperture, 400-μm core optical fiber patch cord (Doric). Emitted fluorescence traveled back through the patch cord, passed through the dichroic, a filter (Semrock, FF01-535/50), and was focused onto a photodetector (Newport, model 2151). The modulated signal passed from the photodetector to a RP2.1 real-time processor (Tucker Davis Technologies) where it was demodulated, and low pass filtered using a corner frequency of 15 Hz. Time-to-live (TTL) pulses denoting the start of behavioral trials were passed to the processor in real time for alignment of calcium recordings to behavioral measures.

### In vitro chloride imaging

Neuro 2A cells (ATCC CCL-131, mycoplasma testing and STR profiling performed by ATCC) were grown in Eagles’ minimum essential medium with 10% FBS (Gibco) at 37°C and 5% CO_2_.

For ICC studies, cells were cultured on 12-mm cover glass (Fisher Scientific, Pittsburgh, PA) coated with fibronectin (Sigma-Aldrich). Cells were transfected with constructs expressing Nb-Ft-TRPV1^Cl−^ or TRPV1^Ca2+^ 24 hours after plating. Cells were stained 48 to 72 hours after transfection.

For imaging studies using the chloride-dependent reporter MQAE, Neuro2A cells were grown on 12-mm cover glass and transfected with plasmids expressing mCherry alone, cotransfected with NbFt-TRPV1^Cl−^ and mCherry, or cotransfected with TRPV1^Ca2+^ and mCherry. Holotransferrin (2 mg/ml; Sigma-Aldrich) was added to cells 24 hours after transfection. Cells were placed at 32°C 24 hours before imaging. Cells were washed three times with Krebs-Hepes buffer (110 mM NaCl, 5.5 mM KCl, 2.5 mM CaCl_2_, 1.25 mM MgCl_2_, 5 mM Hepes, and 10 mM glucose) then incubated with 5 mM MQAE (Invitrogen) in Krebs-Hepes buffer for 1 hour at 37°C. Cells were then washed three times in Krebs-Hepes buffer and placed in glass-bottom dishes with Krebs-Hepes buffer for 15 min before imaging. Imaging was performed using an inverted fluorescence microscope Zeiss Axio Observer Z1 microscope (Carl Zeiss, Oberkochen GER). Cells were imaged every 6 s for a total of 3 min with imaging before and during low AMF treatment with a neodymium magnet (K and J magnetics) to produce a peak field of 230 to 250 mT. Image analysis was performed using FIJI. Briefly, ROIs were selected based on mCherry expression and the mean intensity calculated for each ROI in each image after background subtraction. Chloride responses were quantified as change in fluorescence intensity normalized to baseline fluorescence (Δ*F*/*F*_0_). Imaging was performed on at least three occasions for each condition and chloride responses were measured in 142 cells (Nb-Ft-TRPV1^Cl−^), 76 cells (TRPV1^Ca2+^), and 181 cells (mCherry).

ICC was used to confirm cell surface expression of TRPV1. Live cells were incubated with rabbit anti-TRPV1 (extracellular) polyclonal antibody (Thermo Fisher Scientific, catalog no. PA5-77361) (1:50) in culture medium for 10 min at 37°C followed by five washes with RT culture medium. Cells were then incubated with goat anti-rabbit Alexa Fluor 633 (1:1000) for 10 min at 32°C followed by a further five washes with RT culture medium. Cells were then fixed in 4% PFA in HBSS for 30 min at RT and washed five times in HBSS before mounting using Fluoromount with DAPI (Southern Biotech, Birmingham, AL).

### Imaging acquisition and analysis

Images were taken by an epifluorescence microscope (Olympus BX61 microscope fluorescence microscope fitted with an Olympus DP71 digital camera) or a confocal microscope (Zeiss LSM 900) and analyzed with the ImageJ software (version 1.52p, NIH, United States). For quantification of *c-fos*–positive cells, coronal sections were sampled at intervals of 120 to 160 μm for immunostaining. Three dorsal striatal or nigral sections per mouse were identified using the mouse brain atlas of Paxinos (2007) and scanned bilaterally using a 20x objective. For each selected section, three randomly chosen ROIs with fixed areas were selected for quantification. A Macro Plugin was applied to each section obtained, with a Gaussian blur filter threshold set to 30 and size filter to 20 to remove small objects and low signal cells. *c-fos*–positive neurons were determined only when clear colocalization with DAPI staining was observed. Automatized detection using the ImageJ software in four to five mice per condition (controls versus treated).

### Statistics

To make valid comparisons among experimental groups, mice were randomized based on baseline motor behavior to achieve balanced, unbiased groups. PitX2-Cre mice were randomized based on contralateral rotational counts following systemic apomorphine injection (0.25 mg/kg), measured at 4 weeks after 6-OHDA lesion surgery. The sample size for our experiments was determined by power analysis (*57*), a published work in the field, and previous studies performed by the principal investigators. Exact sample sizes for each experiment are indicated in figure legends. The power was set at 95% probability, effect size to 0.3 to 0.5 depending on the experimental outcome, and significance at *P* < 0.05.

Statistical analyses were conducted using GraphPad Prism 7.0. The statistical test used in each experiment depended on the type of data collected (reported in figure legends). We tested for normality using the Kolmogorov-Smirnov test and Q-Q plots. Two-way ANOVA or two-tailed *t* test for statistical comparisons were performed as appropriate.

For fiber photometry experiments, the change in fluorescence intensity (Δ*F*/*F*) percentage was calculated by subtracting the linear fit of the isosbestic signal to the raw GCaMP signal from the raw GCaMP signal, and the median for the entire recording session was then subtracted from each GCaMP event value and divided by the median; that is, (465_raw_ − 405_fitted_) = 465_net_; (465_net_ − 465_median_)/(465_median_) × 100. Events were computed by comparing means in pre-DMF/DMF/post-DMF or baseline/TMS conditions.

Pearson’s correlation coefficients were used to quantify associations between different readouts (e.g., *c-fos*–positive immunoreactivity versus freezing of gait). When comparing two sets of normally distributed data, unpaired two-tailed *t* tests were used.
