# Supplementary material for: Bidirectional regulation of motor circuits using magnetogenetic gene therapy
Source: Sci Adv. 2024 Oct 9;10(41):eadp9150. doi: 10.1126/sciadv.adp9150 (PMC11463271; doi:10.1126/sciadv.adp9150)
Supplement: Supplementary file 1 — Figs. S1 to S8 Tables S1 and S2 Legends for movies S1 to S5 [file sciadv.adp9150_sm.pdf]

Science Advances
AAAS

Supplementary Materials for

Bidirectional regulation of motor circuits using magnetogenetic gene therapy

Santiago R. Unda *et al.*

Corresponding author: Michael G. Kaplitt, mik2002@med.cornell.edu;
Jeffrey M. Friedman, friedj@rockefeller.edu; Sarah A. Stanley, sarah.stanley@mssm.edu

Sci. Adv. **10**, eadp9150 (2024)
DOI: 10.1126/sciadv.adp9150

The PDF file includes:

Figs. S1 to S8
Tables S1 and S2
Legends for movies S1 to S5

Other Supplementary Material for this manuscript includes the following:

Movies S1 to S5

Supplementary figure 1

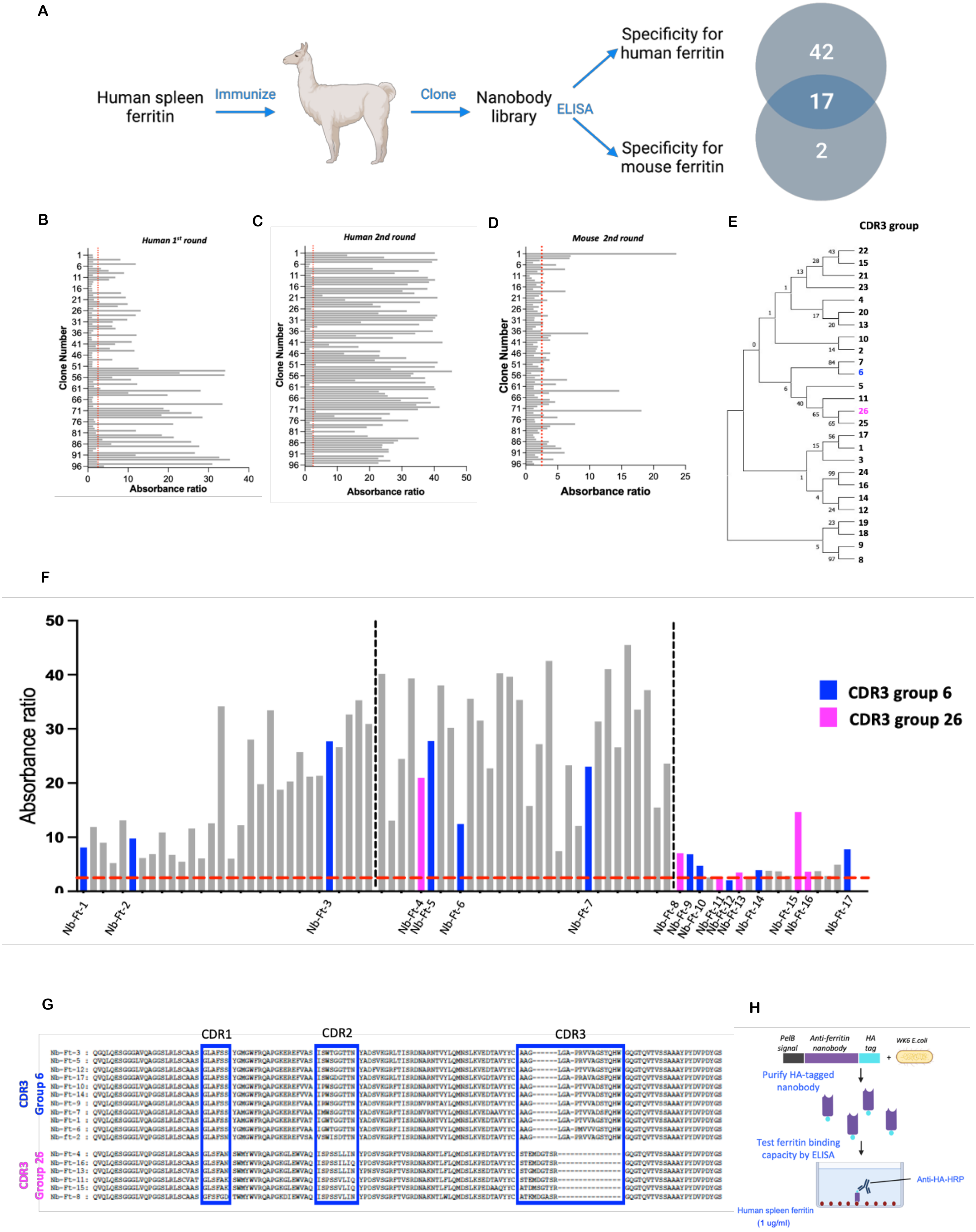

Fig. S1. Generation of Nanobodies (Nb) to human and mouse ferritin. A. Schema of overall method for llama immunization, generation of phage display library, panning for selection of highly reactive nanobody clones to human spleen ferritin and mouse liver ferritin, ELISA and numbers of unique nanobodies reactive to human, mouse or both human and mouse ferritin. Absorbance ratio of nanobody clones for human spleen ferritin (**B, C**) and mouse liver ferritin (**D**). **E**. Classification of nanobody clones based on their similarity on their complementary region 3 (CD3). **F**. Example of ELISA selection for nanobodies from distinct groups for highly reactive clones. Clones were screened after the 1st and 2nd rounds of panning against human ferritin and after the 2nd round of panning against mouse ferritin. Clones exhibiting absorbance ratio greater than 2.5 (dashed red line) were selected for further characterization. **G**. Alignment of amino acid sequence of nanobodies belonging to CDR3 groups 6 and 26 with the highest reactivity to human and mouse ferritin. CDR1, CDR2, and CDR3 are colored in blue. The nanobodies' CDR3 group and names are on the right. **H**. Schema of method for quantification of nanobody binding to human spleen ferritin. HA-tagged nanobodies from CDR3 groups 6 and 26 were purified from transformed WK6 E. coli by metal-ion affinity chromatography.

Supplementary figure 2

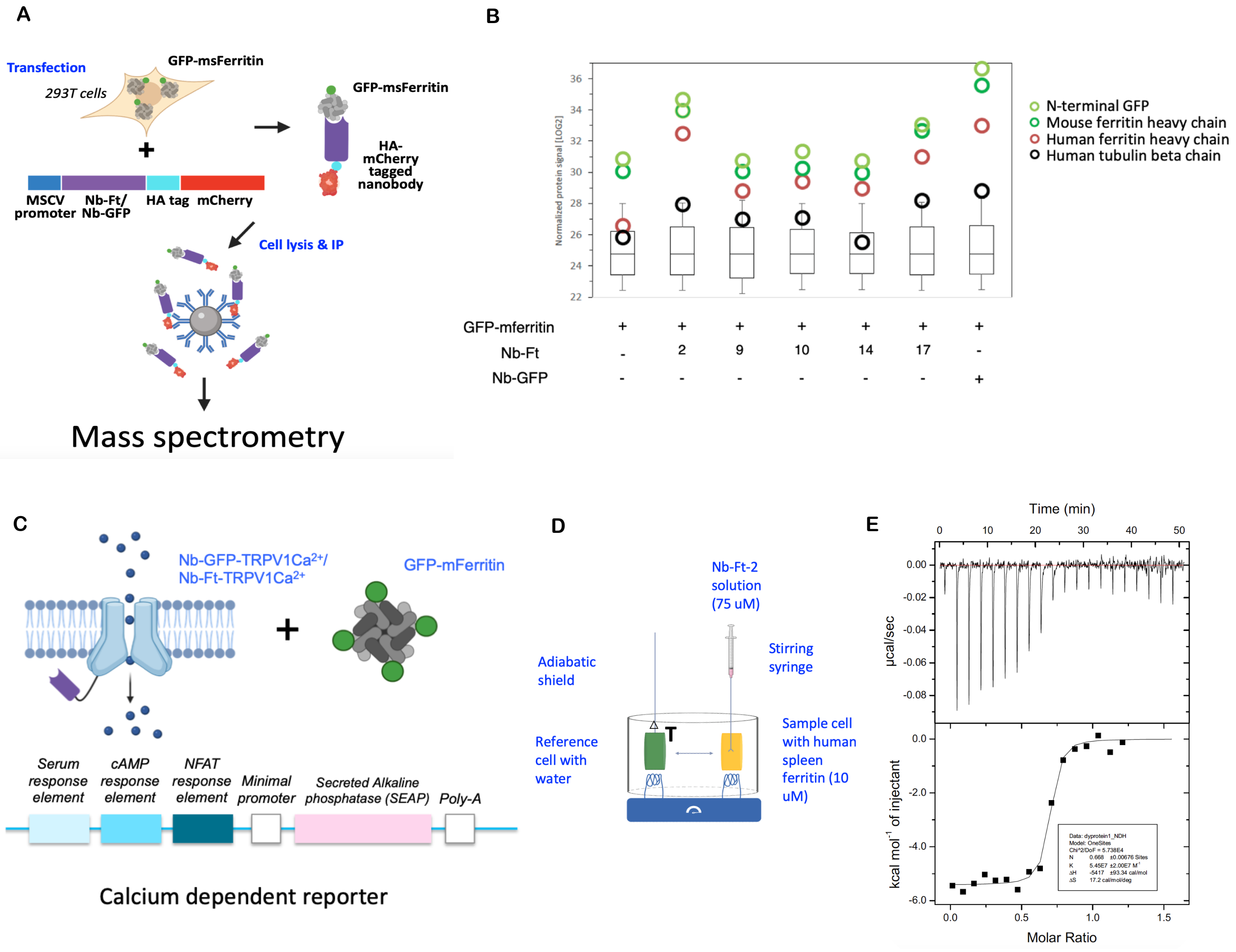

Fig. S2. Screen of Nb clones to mouse ferritin. A. Schema of method to characterize Nb-Ft binding to mouse ferritin. Mammalian expression plasmids with Nb-Ft sequences (Nb-Ft-2, -9, -10, -14, -17) or Nb-GFP fused to HA-tagged mCherry were co-transfected into HEK-293T cells along with a plasmid expressing GFP-tagged mouse ferritin (GFP-mFerritin). After 48 hours, cells were lysed and HA-tagged nanobodies along with their bound proteins were immunoprecipitated using anti-HA conjugated agarose beads. Eluted proteins were examined by SDS-PAGE and mass spectrometry. **B.** Quantification of nanobody-bound proteins by mass spectrometry normalized for protein signal (log2). The protein signals for mouse ferritin heavy chain (transfected as GFP-mFerritin), human ferritin heavy chain (FTH1_human, endogenous to HEK-293T cells), and N-terminal GFP moiety of GFP-mFerritin are shown along with a control peptide, human tubulin (TBB5_human). The overall protein signal shown in the box plot does not differ between test conditions. **C.** Schema of method to validate ability of Nb-ferritin clones to transduce magnetic field into cell activation. Constructs with Nb-Ft sequences (Nb-Ft-2, -9, -10, -14, -17) fused to the N-terminal of TRPV1^{Ca2+} were generated and transfected into HEK-293T cells along with a plasmid expressing GFP-tagged mouse ferritin (GFP-mFerritin) and a calcium-dependent secreted alkaline phosphatase (SEAP). Cells transfected Nb-GFP-TRPV1^{Ca2+}/GFP-mFerritin and the reporter were used as a positive control. **D.** Schema of isothermal titration calorimetry assay for binding of anti-ferritin nanobody-2 (Nb-Ft2) to human spleen ferritin. Human spleen ferritin (10 μM) was titrated against 75 μM Nb-Ft2 (75 μM, single 0.4 μL injection, then 19 injections of 2 μL each, 150-sec intervals, 750 rpm stirring) with a reference power of 10 μcal/sec. **E.** Binding affinity and interaction between Nb-Ft2 and human spleen ferritin by isothermal titration calorimetry at 25°C (K_d = 0.54 ± 0.2 μM).

Supplementary figure 3

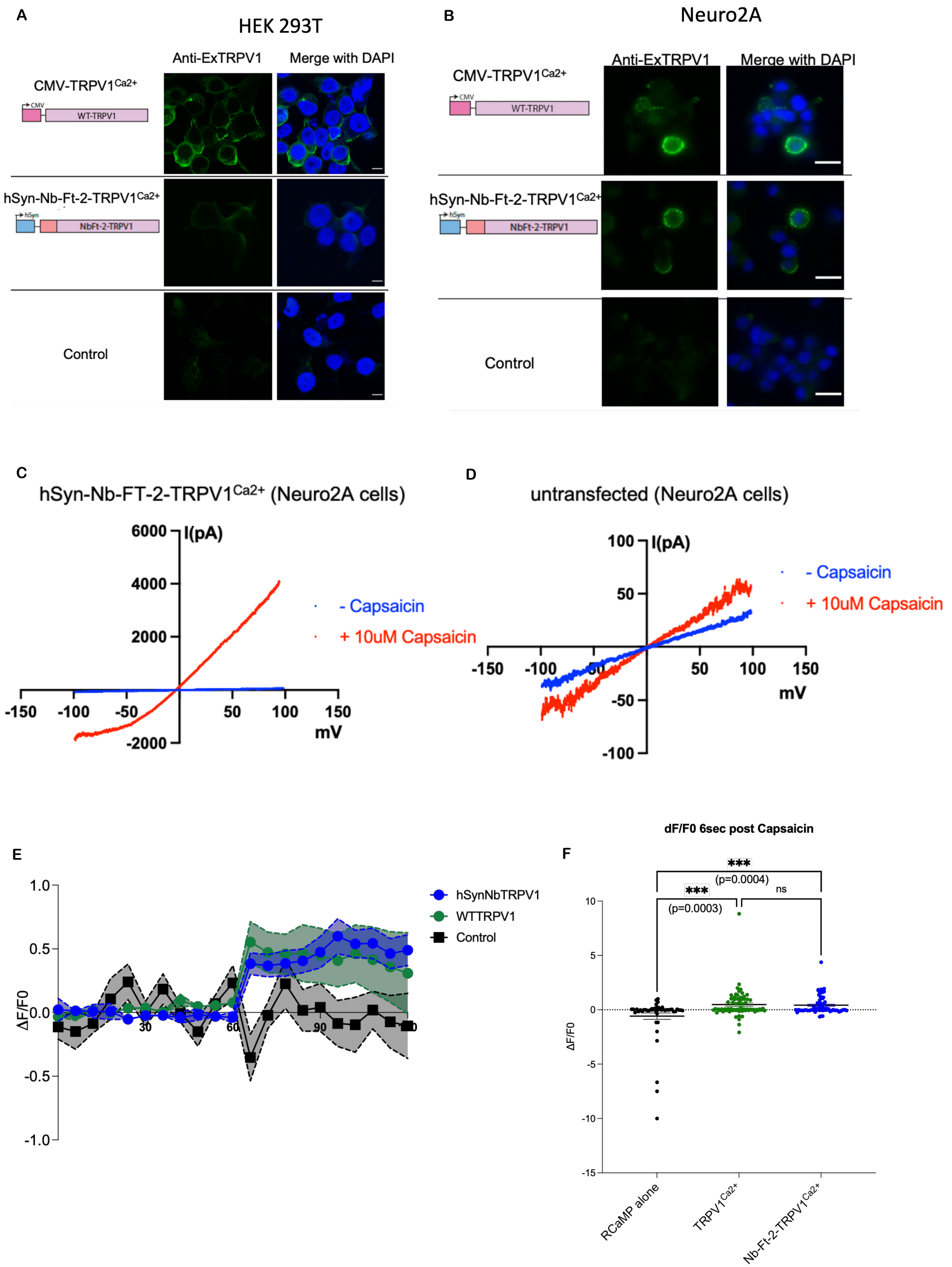

Fig. S3. Functional validation of Nb-Ferritin clones *in vitro.* Cell surface expression of Nb-Ft-2-TRPV1^{Ca2+} by immunolabeling using antibodies directed to the extracellular portion of TRPV1 in transfected human HEK-293T (**A**) and murine Neuro2A cells (**B**) without permeabilization. Capsaicin-dependent currents in pulled patches from Neuro2A cells transfected with the hSyn-Nb-Ft-2-TRPV1^{Ca2+} construct (**C**) and untransfected cells (**D**). **E**. Changes in RCaMP fluorescence normalized to baseline fluorescence ($\Delta F/F_0$) with capsaicin treatment of HEK-293T cells expressing RCaMP alone (49 cells), TRPV1^{Ca2+} (65 cells) or Nb-Ft-2-TRPV1^{Ca2+} (73 cells). Error bars represent mean +/-SEM. **F**. Peak $\Delta F/F_0$ with capsaicin treatment of HEK-293T cells expressing RCaMP alone (49 cells), TRPV1^{Ca2+} (65 cells) or Nb-Ft-2-TRPV1^{Ca2+} (73 cells). Data were analyzed by ordinary one-way ANOVA with Tukey's multiple comparison test, **** $p < 0.0001$. Error bars represent mean ± SD.

Supplementary figure 4

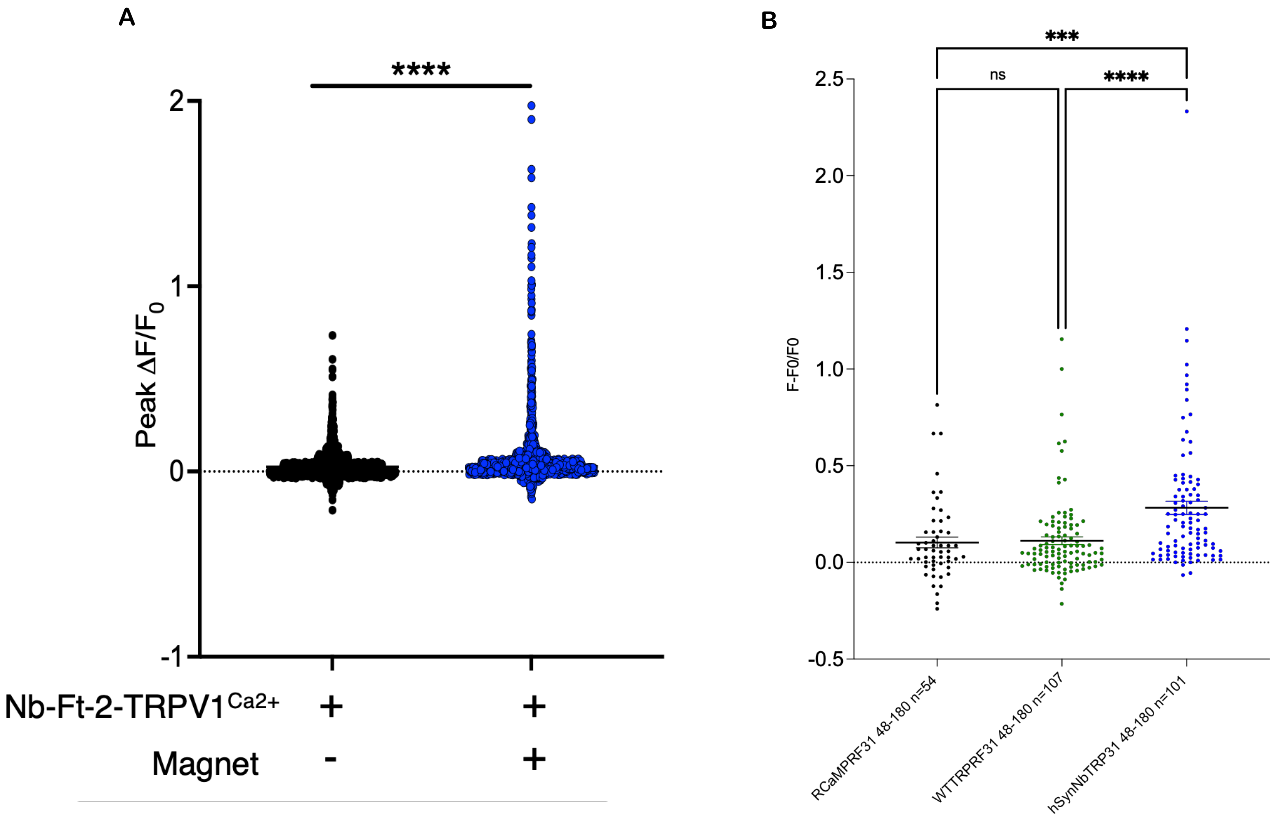

Fig. S4. Peak fluorescence in response to magnet treatment. A. Peak $\Delta F/F_0$ of Neuro2A cells expressing Nb-Ft-2-TRPV1^{Ca2+} with (786 cells) or without (1659 cells) magnet treatment. Data were analyzed by Mann Whitney U test **** $p < 0.0001$. **B.** Peak $\Delta F/F_0$ with magnet treatment of HEK-293T cells expressing RCaMP alone (54 cells), TRPV1^{Ca2+} (107 cells) or Nb-Ft-2-TRPV1^{Ca2+} (101 cells). Data were analyzed by ordinary one-way ANOVA with Tukey's multiple comparison test, **** $p < 0.0001$. Error bars represent mean ± SD.

Supplementary figure 5

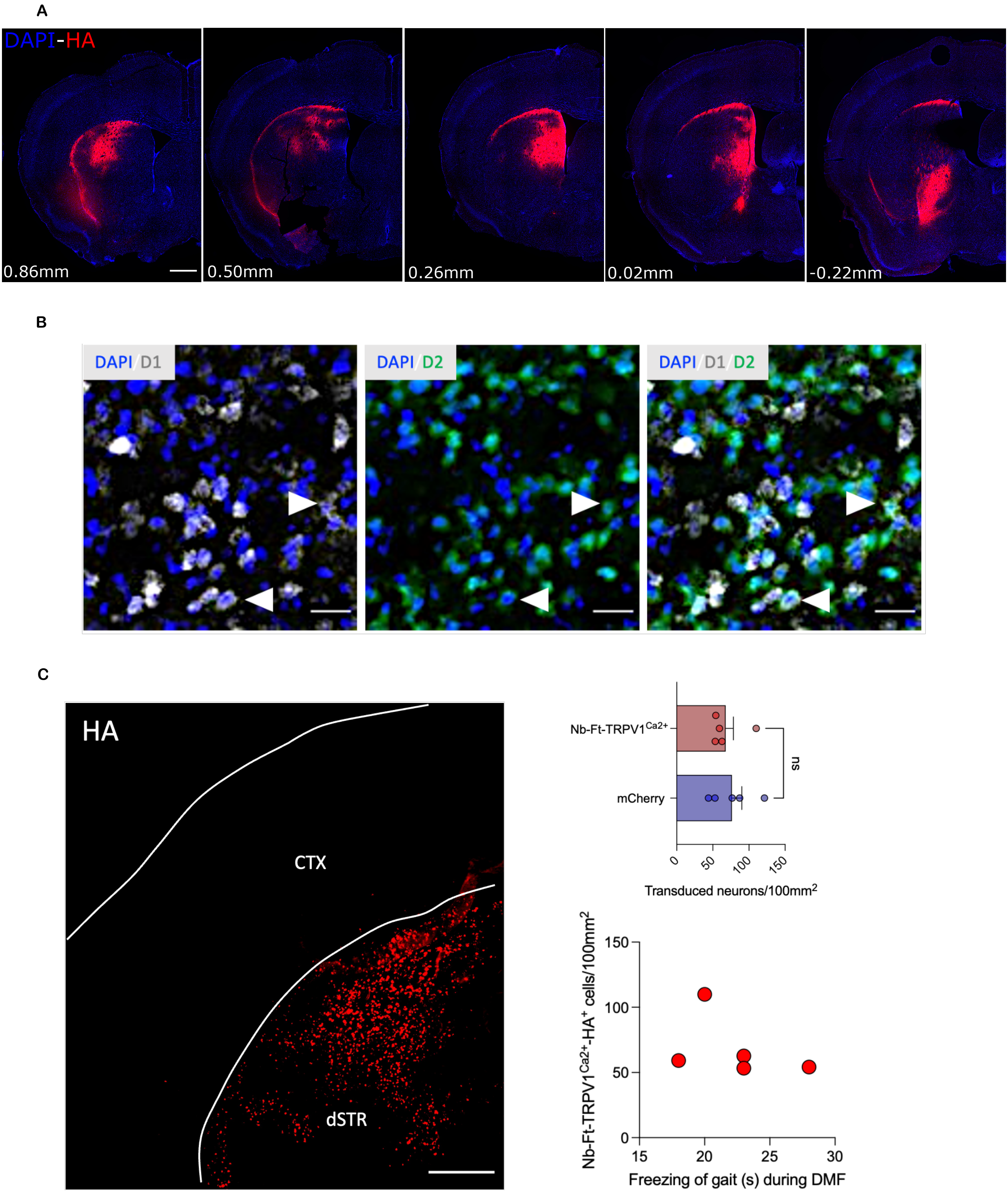

Fig. S5. Viral-mediated expression of Nb-Ft-TRVP1^{Ca2+} in striatal iSPNs elicit parkinsonian motor behavior. A. Rostro-caudal expression pattern across the dorsal STR following AAV injections. **B**. RNA probe validation for D1 and D2 neurons**. C.** Example of Nb-Ft-TRVP1^{Ca2+} expressing mouse stained for HA (left), number of transduced neurons in STR between mCherry and Nb-Ft-TRPV1Ca2+ groups (upper right), and correlation of viral transduction with freezing of gait time during DMF (lower right).

Supplementary figure 6

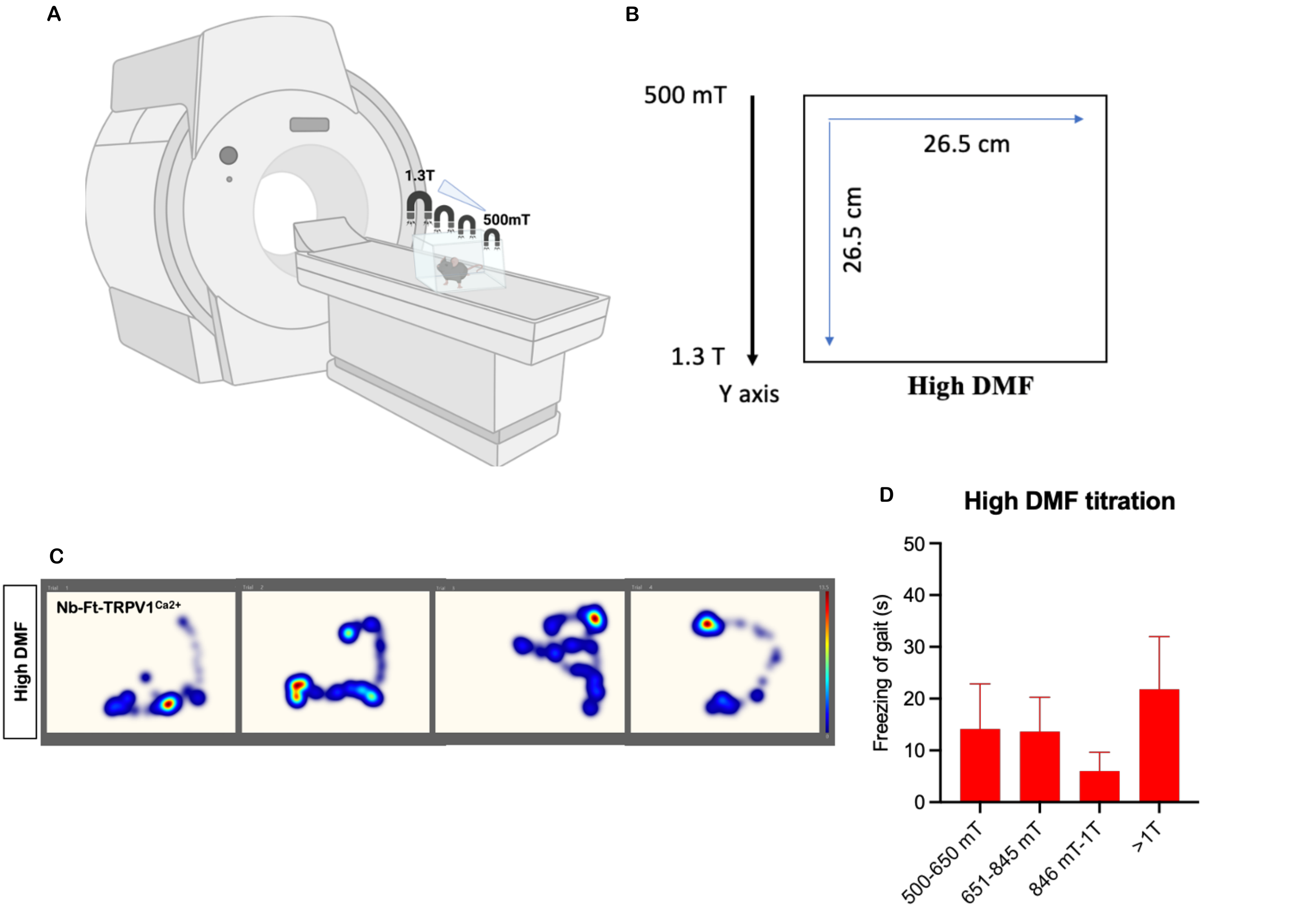

Fig. S6. High DMF Titration in Nb-Ft-TRPV1^{Ca2+} expressing mice. A. Schema for high direct magnetic field (DMF) titration. **B.** Magnetic field mapping in the "y" axis of the cage used for behavioral assessment. **C.** Individual activity heatmap example of motor activity during bilateral striatal High DMF titration in Nb-Ft-TRPV1^{Ca2+} expressing mouse. **D.** Freezing of gait in different magnetic field gradients in the High DMF titration ranges (red bars, n=5) in Nb-Ft-TRPV1^{Ca2+} expressing mice.

Supplementary figure 7

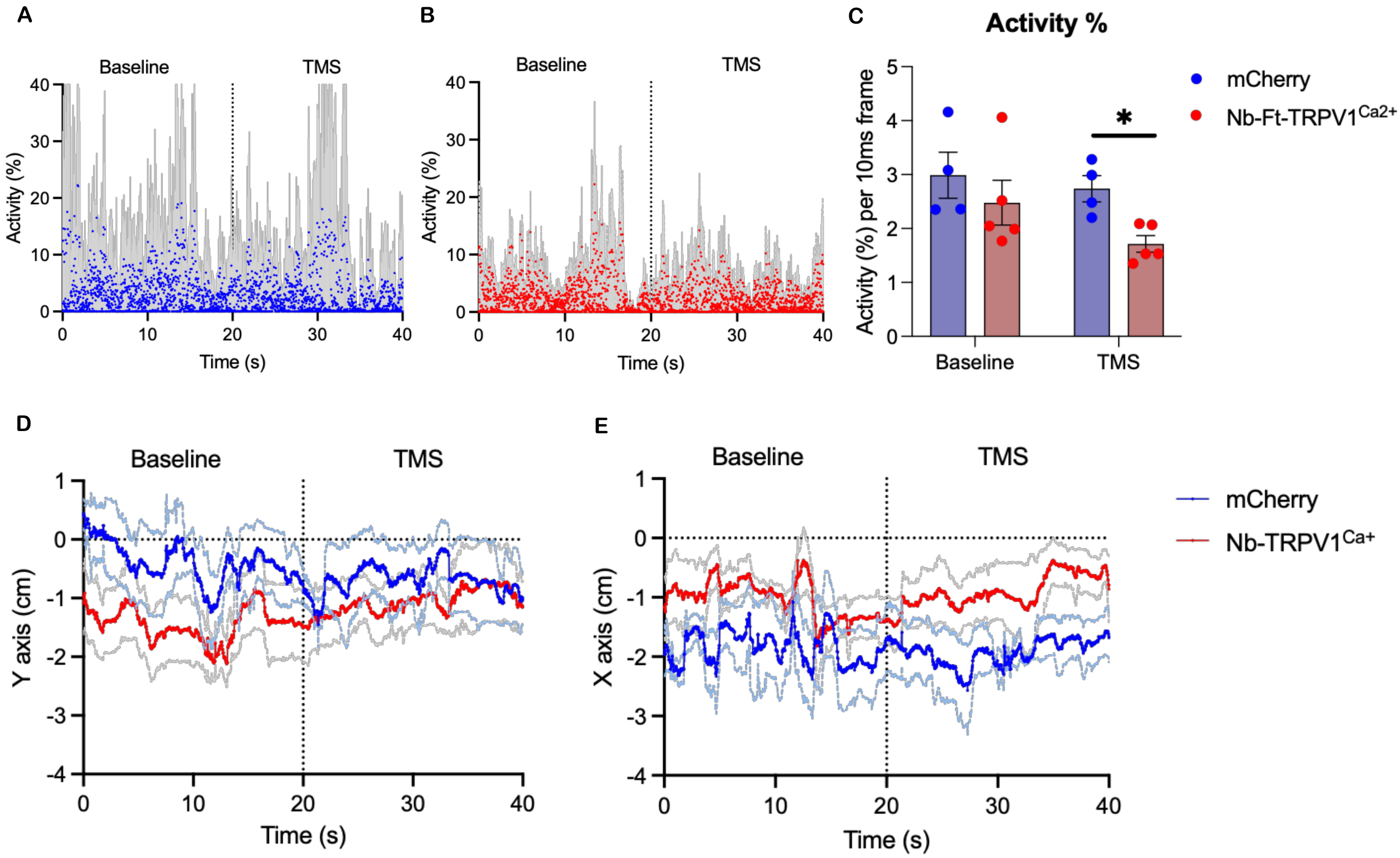

Fig. S7. Transcranial magnetic stimulation (TMS) treatment alters motor behavior in Nb-Ft-TRPV1^{Ca2+} expressing mice. Activity percentage (%) during baseline (left) and TMS (right) treatment in the **A.** mCherry group (blue), and **B.** Nb-Ft-TRPV1^{Ca2+} group (red). **C.** Average distance during baseline and TMS treatment in mCherry (blue, n=4) and Nb-Ft-TRPV1^{Ca2+} (red, n=5) groups. Error bars show SEM. * represents p value < 0.05 with Student's t test. Tracking data of change of position in the **D.** "y" axis, and **E.** "x" axis in mCherry (blue), and Nb-Ft-TRPV1^{Ca2+} group(red) during baseline and TMS treatment.

Supplementary figure 8

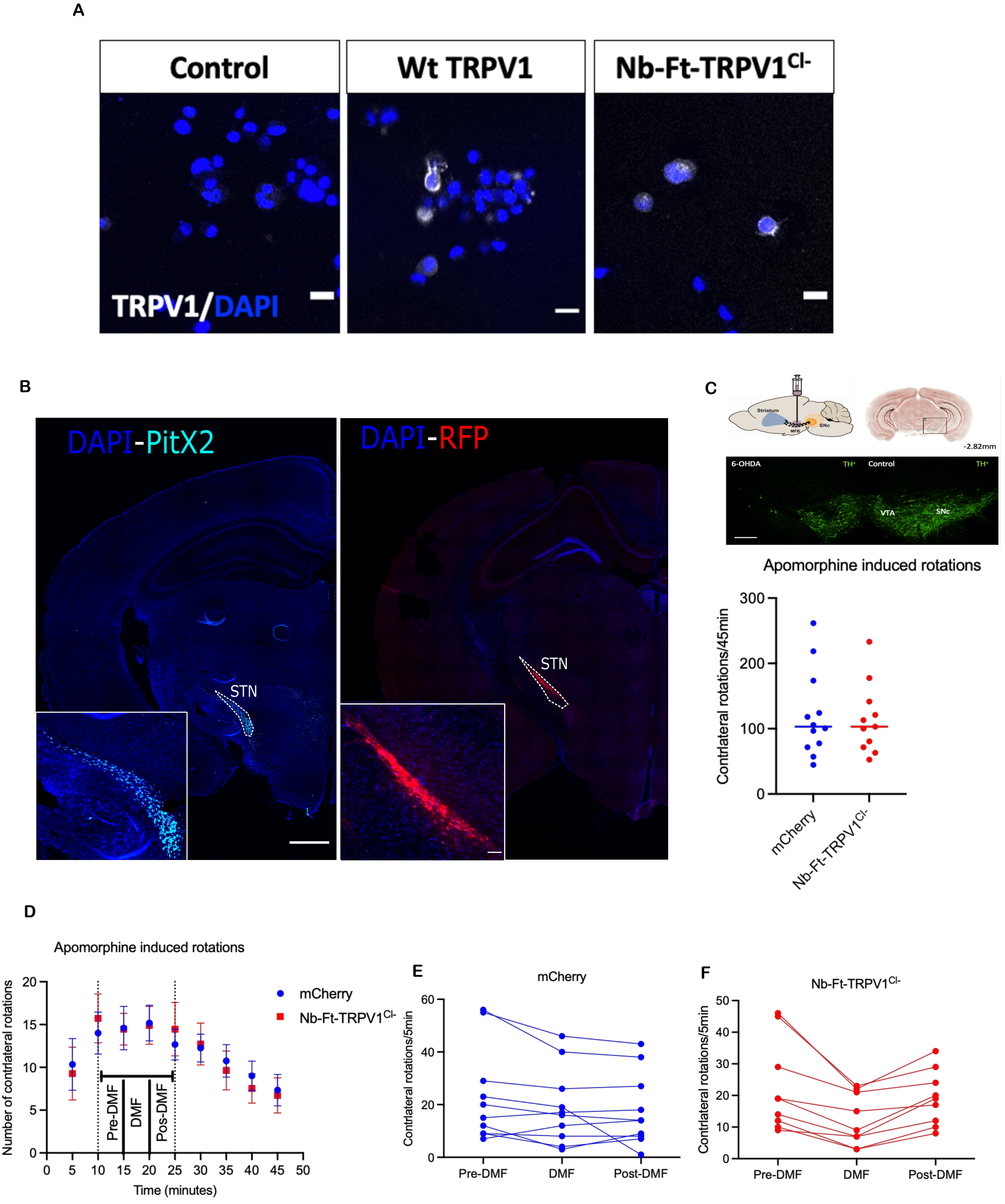

Fig. S8. Application of mutant Nb-Ft-TRPV1^{Cl-} membrane channel for subthalamic nucleus in mouse model of Parkinson disease. **A.** Immunostaining for cell surface expression of TRPV1 in Neuro2A cells transfected with mCherry, TRPV1^{Ca2+} or Nb-Ft-TRPV1^{Cl-} (from left to right). Scale bar 20um. **B.** Representative RNAscope staining for PitX2+ cells in the STN and representative immunostaining of RFP+ positive cells in PitX2-cre mice injected with the AAV-DIO-mCherry vector in the STN. **C**. Immunostaining shows unilateral loss of TH+ neurons in the substantia nigra (SN) of lesioned PitX-2-Cre mice with 6-OHDA in the MFB **and** randomization 6-OHDA lesioned mice based on apomorphine induced rotations. **D.** Selection of time points for behavioral testing during DMF application. Individual variation in the number of contralateral rotations for 5 minutes during pre-DMF, DMF, and post-DMF exposure in the **E.** mCherry group (n=10), and **F.** Nb-Ft-TRPV1^{Cl-} group (n=9).

	GFP-mferritin	Nb-Ft 2	Nb-Ft 9	Nb-Ft 10	Nb-Ft 14	Nb-Ft 17	Nb-GFP
	Normalized protein signal (Log2)						
**Human ferritin heavy chain**	26.6	32.5	28.8	29.4	29.0	31.0	33.0
**Mouse ferritin heavy chain**	30.1	34.0	30.1	30.3	30.0	32.7	35.6
**N-terminal GFP**	30.9	34.7	30.8	31.4	30.8	33.1	36.6
**Human tubulin beta chain**	25.9	28.0	27.1	27.1	25.6	28.2	28.9
**Human ferritin enrichment (compared to GFP-mferritin alone)**		58.1	4.6	7.0	5.1	20.9	82.6
**Mouse ferritin enrichment**		14.5	1.0	1.1	0.9	6.0	44.3

Table S1. Nanobody-ferritin clones mouse ferritin enrichment.

ID	NET Rotations				Change from pre-MRI		Change post-DMF relative to DMF
	Pre-MRI	MRI	Pos-MRI	Group	MRI	Post-DMF	Post-DMF
17	56	40	38	mCherry	-28.571429	-32.142857	-5
9	12	3	9	mCherry	-75	-25	200
19	9	4	7	mCherry	-55.555556	-22.222222	75
22	23	19	1	mCherry	-17.391304	-95.652174	-94.73684211
4	46	22	34	TRPV1	-52.173913	-26.086957	54.54545455
21	19	9	20	TRPV1	-52.631579	5.26315789	122.2222222
7	12	3	10	TRPV1	-75	-16.666667	233.3333333
10	29	26	27	mCherry	-10.344828	-6.8965517	3.846153846
248	10	3	8	TRPV1	-70	-20	166.6666667
250	*14*	*7*	*12*	*TRPV1*	-50	-14.285714	71.42857143
20	*19*	*15*	*17*	*TRPV1*	-21.052632	-10.526316	13.33333333
28	*9*	*8*	*8*	*mCherry*	-11.111111	-11.111111	0
13	55	46	43	mCherry	-16.363636	-21.818182	-6.52173913
14	45	23	29	TRPV1	-48.888889	-35.555556	26.08695652
15	20	16	14	mCherry	-20	-30	-12.5
192	*15*	*17*	*18*	*mCherry*	13.3333333	20	5.882352941
168	29	21	24	TRPV1	-27.586207	-17.241379	14.28571429
194	9	7	19	TRPV1	-22.222222	111.111111	171.4285714
23	7	12	14	mCherry	71.4285714	100	16.66666667

Table S2. Apomorphine-induced rotations in PitX2-Cre mice. Raw data of contralateral rotations and percentage of change from Pre-MRI in 6-OHDA PitX2-Cre mice expressing Nb-Ft-TRPV1Cl- (TRPV1) or control (mCherry) in the STN during Pre-MRI/MRI and Post-MRI conditions.

Supplementary videos/movies

Main text

Supplementary movie 1
Open field video tracking. Representative Nb-Ft-TRPV1^{Ca2+} mouse during DMF “ON” and DMF “OFF” conditions.

Supplementary movie 2
Open field video tracking. Representative DMF “ON” condition in mCherry control mouse.

Methods (TMS section)

Supplementary movie 3
TMS Method machine capacity. Increasing capacity set up to achieve magnetic-field induced phenotype in Nb-Ft-TRPV1^{Ca2+} mice.

Supplementary movie 4
TMS Method machine capacity. Representative mCherry control mouse responding to 20% capacity.

Supplementary movie 5
TMS Method pain/twitching threshold. Electric-shock-induced responses (twitching) when TMS machine capacity was set up at 25%.
